# Insidious systems: a scoping review of Black youth and the criminal justice system in Canada

**DOI:** 10.1186/s40352-026-00396-2

**Published:** 2026-01-15

**Authors:** Joshua E Yusuf, Gervin Apatinga, Barbara-Ann Hamilton-Hinch, Annette Bailey, W. Andy Knight, Geoffrey Maina, Aloysius Nwabugo Maduforo, Bukola Salami

**Affiliations:** 1 Dalhousie University, Halifax, Canada; 2 University of Calgary, Calgary, Canada; 3 Toronto Metropolitan University, Toronto, Canada; 4 University of Alberta, Edmonton, Canada; 5 University of Saskatchewan, Saskatoon, Canada

**Keywords:** Black youth, Criminal justice, Racial injustice, Anti-Black racism

## Abstract

**Introduction:**

A growing concern within and beyond Black communities is the increasing injustices faced by Black children and youth, especially the indelible injustices imposed in their involvement with the Canadian criminal justice system. This study conducts a scoping review of the existing literature on Black youth involvement with the Canadian criminal justice system.

**Methods:**

The scoping review followed Arksey and O’Malley’s five step framework and adhered to the Preferred Reporting Items for Systematic Reviews and Meta-Analyses extension for Scoping Reviews guidelines. We broadly searched seven databases for studies focusing on Black children and youth (0–30 years) in Canada. This scoping review reports data extracted from 35 criminal-justice related studies.

**Results:**

Research on Black youth and the criminal justice system in Canada has grown since 2000, with most studies (60%) focusing on Ontario. Findings were categorized by systems (child welfare, education, and health) and their interactions with the criminal justice system. Black youth’s contact with the criminal justice system is an extension of their daily interactions and experiences within other systems, where decisions, actions, and culture are underscored by processes of racial injustice.

**Conclusions:**

While there is more attention to research on Black youth involvement with the Canadian criminal justice system, more work is needed to understand their experiences at different levels of involvement with the Canadian criminal justice system, in order to guide and advance the development of relevant policies, strategies, and interventions. Focused efforts should be directed at addressing anti-Black racism within Canadian systems, particularly in child welfare, healthcare, and education, due to their deep and troubling connection to the criminal justice system.

**Supplementary Information:**

The online version contains supplementary material available at 10.1186/s40352-026-00396-2.

## Insidious systems: a scoping review of Black youth and the criminal justice system in Canada

Within the global sphere, Canada is known as a leader in health promotion, a champion in the pursuit of human development, and an advocate for traditions and practices that uphold equity, diversity, and inclusion (United Nations General Assembly, 2015; Weldon & Hoffman, 2021). While this should indicate a distinguished and progressive trend in Canada’s efforts against systemic inequalities, evidence still point to the troubling entrenchment of racial discrimination, primarily among Black Canadians, across multiple institutions, including the criminal justice system, education, and healthcare (Alhassan et al., 2024; Husbands et al., 2022; Williams et al., 2024). In 2019, 41% of Black Canadians reported experiencing discrimination, and by 2022, this number rose to 53% (Cotter, 2022; Department of Canadian Heritage, 2024). This pattern reflects a long history of colonial legacies, systemic oppression, and racial profiling that disproportionately impact Black communities in Canada (Dryden & Nnorom, 2021).

A growing concern within Black communities is the disproportionate criminalization of Black children and youth. The overrepresentation of Black youth in the criminal justice system has steadily increased, rising from 8% in 2002 to 19% in 2006 and reaching 29% by 2020 (Samuels-Wortley, 2022; Statistics Canada, 2015).Nearly half of Black individuals in the justice system are under 30 years old, highlighting the early interference of injustice in the life trajectory of Blacks, and the urgent need for early and systemic interventions at different stages of development (Government of Canada Office of the Correctional Investigator, 2013). Identified factors contributing to this overrepresentation include systemic anti-Black racism, the school-to-prison pipeline (STPP), over-policing and stop-and-search practices, poverty, and inadequate services (Department of Justice Government of Canada, 2022; Maynard, 2017; Samuels-Wortley, 2022). Previous scholarship demonstrates that Black boys/youth experience disproportionately higher rates of school suspension, police brutality, racial profiling, micro-aggressions, witnessing and/or losing multiple loved ones to gun violence, and are more likely to be randomly stopped-and-searched by the police compared to their white counterparts (Bailey et al., 2024; Department of Justice Government of Canada, 2022; Maynard, 2017; Wortley, 2019; Wortley & Jung, 2020). These highly race-based experiences are heavily rooted in systemic injustices and have far reaching and rippling impacts on Black youths’ experiences with complex trauma, heightened anxiety, and hypervigilance (Bailey et al., 2024). Evidence shows that these racial trauma experiences alters Black youth’s identity and their worldview, and exacerbate feelings of marginalization, exclusion, isolation, all of which can result in lower educational attainment, lower socioeconomic status, family disruptions, and overall negative health and well-being (Bailey et al., 2024; Boateng & Regragui, 2023; Department of Justice Government of Canada, 2014; Jennifer White, 2021; Ontario Human Rights Commission, 2018; Walker, 2022). Taken together, these experiences have not only created disproportional mental health challenges for Black youth, but their combined effects have also perpetuated and translated Black youth’s experiences with the juvenile and criminal justice systems (Bailey et al., 2024; Bernard & Smith, 2018). The urgency for enhanced policies and programs to address systemic anti-racism and reduce the overrepresentation of Black youth in the Canadian criminal justice system, is therefore, predicated on the multiple and connected racial experiences that continue to press on the well-being of Black youth and their families.

While evidence underscores that Black Canadian youth are disproportionately involved with the criminal justice system, leading to negative health and social consequences, there remains a lack of extensive research on this crucial issue in Canada. To effectively address systemic anti-Black racism and the overrepresentation of Black youth in the criminal justice system, more needs to be known about this issue to engage governments, policymakers, and non-governmental organizations in developing effective and impactful interventions. In an epoch where world leaders have committed to the principle of leaving no one behind in the Sustainable Development Agenda, it is essential to explore the overrepresentation of Black youth in the Canadian criminal justice system to guide policies and programs aimed at addressing the broader health and social challenges faced by Black Canadian youth.

Given the urgency of this issue and the need for evidence-informed policy responses, this study conducts a scoping review of the existing literature on Black youth involvement with the Canadian criminal justice system. The review aims to s ynthesize findings, identify knowledge gaps, and provide a foundation for future research and policy interventions. By exploring the intersections of race, law enforcement, education, healthcare, and social systems, this scoping review seeks to contribute to the growing body of knowledge that informs anti-racist policies and equitable justice practices.

## Methods

This scoping review emerged as a directive from the establishment of a national collaborative project ‘Transforming the Lives of Black Children and Youths in Canada’with emphasis on five thematic areas (health, education, criminal justice, child welfare and migration). The collaborative advised on the broad research question to be addressed. While all five thematic areas were included in the scoping review, this paper reports on the review of the criminal justice theme. The criminal justice system was defined in alignment with the Government of Canada’s definition that states “The criminal justice system apprehends, prosecutes, defends, sentences, rehabilitates, and reintegrates those who are accused or convicted of illegal activity” (Department of Justice, 2024, para. 1). This scoping review followed Arksey and O’Malley’s (2005) framework that consists of a five-stage methodology: identifying the research question; identifying relevant studies; study selection; charting the data; and collating, summarizing, and reporting the results. Data were reported in accordance with the Preferred Reporting Items for Systematic Reviews and Meta-Analyses extension for Scoping Reviews (PRISMA-ScR)(Tricco et al., 2018).

### Identifying relevant studies

#### Data sources and search strategy

The search was conducted in May 2024, in seven databases: Ovid MEDLINE(R) ALL, Embase, APA PsychInfo, Academic Search Complete, SocINDEX with full text, Web of Science, and SCOPUS. These databases were selected for their extensive coverage and relevance to the study’s thematic areas. The search strategy consisted of terms considered by the research team to capture the breadth of research related to the purpose of Transforming the Lives of Black Children and Youths and the five thematic areas. That is, the search strategy was inclusive of all five themes areas relevant to this purpose, including the criminal justice theme. Approaching the scoping review through a broad search strategy allowed for mapping of the literature on Black children and youth in Canada to address the projects overarching objectives, ensured efficient use of research resources and reduced redundancies.

#### Citation management

All citations were uploaded into Covidence, an online software used for managing review studies. Covidence automatically removes duplicate citations in the initial importing process. Duplicates that were found to have bypassed the software (due to variations in citation information imported) were removed manually when found.

#### Eligibility criteria

Studies were eligible for inclusion if they were (i) a research paper, (ii) focused on Black populations (iii) the participants resided in Canada, and (iv) have a focus on social, economic, health and/or any other aspect of wellbeing of a child or youth. To be included in this scoping review, a research paper must have included a method, methodology, and data collection (including through interviews, survey, focus groups, intervention, document analysis, artifact, secondary analysis, etc.). Black populations included historical/generational African Canadians, as well as immigrants from countries of African or Caribbean descent and/or from countries that are predominantly Black. The criteria for residing in Canada were not limiting and included those with citizenship, permanent residency, temporary foreign workers, refugees, and/or undocumented persons. Studies were excluded if they were reviews (e.g., systematic reviews, literature reviews, scoping reviews); focused on the African countries Algeria, Egypt, Libya, Morocco, Tunisia, or Morocco; and/or did not focus on child health (e.g., a study that focused on single mothers’ well-being but did not describe the child). The research team noted all review studies, and three member of the research team conducted reference chaining for studies that would meet the inclusion criteria.

### Study selection

#### Title and abstract relevance screening

Across all thematic areas, a total of 8,078 studies were uploaded into Covidence; and 5,427 studies, after duplicate removal, underwent title and abstract screening based on the inclusion and exclusion criteria. Two independent reviewers assessed the eligibility of each study. Any disagreement between these reviewers was resolved by a third reviewer who was a senior member of the research team.

#### Full text screening

**Fig. 1:**
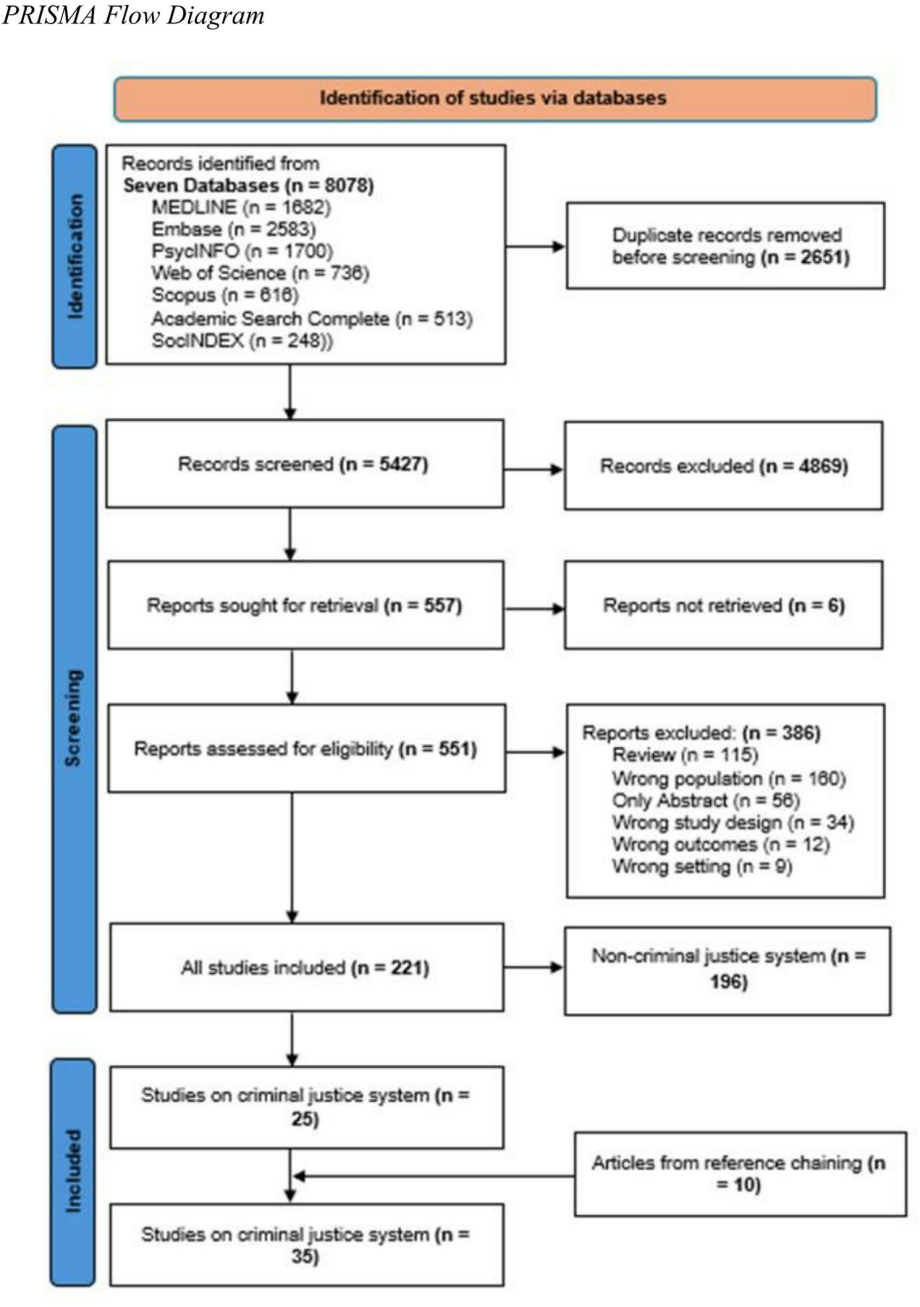
PRISMA Flow Diagram

Across all thematic areas, a total of 557 studies were deemed relevant following the completion of title and abstract screening and underwent full text screening. Two independent reviewers read the full text of each article to assess their alignment with inclusion criteria. Any disagreement between these reviewers was resolved by a third reviewer who was a senior member of the research team. Reviewers made note of review studies so that they could undergo reference chaining by four members of the research team. A total of 35 articles related to the criminal justice thematic area were included for data extraction, 25 articles were through database search while 10 articles were through reference chaining (See Fig. 1 for more details). The included articles are listed on the data extraction file (supplementary file 1). Following the initial full text screening, articles were screened according to identified thematic areas for the ‘Transforming the Lives of Black Children and Youths in Canada’ project.

As this scoping review is focused on the criminal justice thematic area, components of the criminal justice system included legislatures, law enforcement, legal services and courts, victim services, correctional services. Furthermore, studies that focused on other systems (e.g., education or health) but had findings directly related to the criminal justice system were considered for inclusion in this paper (e.g., results that discussed police involvement in schools). The government of Canada recognizes the interrelated nature of the criminal justice system with other systems and therefore we adopted this recognition. We only report findings from these studies that relate to the criminal justice system.

Two independent reviewers screened the full texts for a connection to the criminal justice system. Initial discrepancies were discussed with senior members of the research team on two separate occasions. These discussions resulted in one member of the research team reconducting screening for alignment with the criminal justice system definition. A third reviewer conducted full text screening of a subset of articles (those identified from reference chaining). No further discrepancies were identified following these corrections.

#### Data extraction

Data extraction was completed by one member of the research team and reviewed by three senior members of the research team for accuracy. The following fields were extracted: author(s), title, year of publication, province of study, study design, data collection source (e.g., Black youth and children, parents, service providers), sample size, child and youth age range, gender, country of origin, research objectives/research questions, data collection methods, key findings, policy and practice implications, and research implications. The extraction process was managed using Microsoft Excel (supplementary file 1).

## Results

Figure 1 presents A PRISMA flow diagram outlining the study selection process. After reviewing the files for applicability to the criminal justice system, 35 studies met inclusion criteria for this scoping review (Table or supplementary file 1) (Adjei et al., 2018; Adjei & Minka, 2018; Alaazi et al., 2018; Antwi-Boasiako et al., 2021, 2022a, b; Archie et al., 2010; Bailey et al., 2024; Beausoleil et al., 2017; Boatswain-Kyte et al., 2024; Cénat et al., 2023; Clarke, 2011, 2012; Creese, 2019; Desruisseaux et al., 2002; Edwards et al., 2023; Ellis et al., 2015; Hayle et al., 2016; Huculak & Peckmann, 2012; Khalil et al., 2021; King et al., 2017; Knight et al., 2022; Litchmore, 2022; Manzo & Bailey, 2005; Mason et al., 2022; Millar, 2010; Ngobi et al., 2020; Noorishad et al., 2023; Osman et al., 2024; Owusu-Bempah et al., 2023; Ruck & Wortley, 2002; Sibblis et al., 2022; Syed et al., 2018; Waldron et al., 2023; Wortley & Tanner, 2005).

### Characteristics of included studies

Table 1 provides an overview of the 35 included studies. Of these, a slight majority of studies used a qualitative approach (*n* = 20) (Adjei et al., 2018; Adjei & Minka, 2018; Alaazi et al., 2018; Antwi-Boasiako et al., 2022a, b; Bailey et al., 2024; Cénat et al., 2023; Clarke, 2011, 2012; Creese, 2019; Edwards et al., 2023; Khalil et al., 2021; Litchmore, 2022; Manzo & Bailey, 2005; Mason et al., 2022; Ngobi et al., 2020; Noorishad et al., 2023; Osman et al., 2024; Syed et al., 2018; Waldron et al., 2023). A majority of studies were geographically focused on Ontario (*n* = 21Antwi-Boasiako et al., 2021, 2022a, b; Archie et al., 2010; Bailey et al., 2024; Beausoleil et al., 2017; Cénat et al., 2023; Clarke, 2011, 2012; Edwards et al., 2023; Ellis et al., 2015; Hayle et al., 2016; Khalil et al., 2021; King et al., 2017; Litchmore, 2022; Ngobi et al., 2020; Noorishad et al., 2023; Owusu-Bempah et al., 2023; Ruck & Wortley, 2002; Syed et al., 2018; Wortley & Tanner, 2005).

**Table 1:** Characteristics of included studies (*n* = 37)

Outcome	*N* (%)
Research approach	
Qualitative	20 (57.1)
Quantitative	15 (42.9)
Province/Territory	
Ontario	21 (60.0)
Alberta	4 (11.4)
Quebec	3 (8.6)
Nova Scotia	2 (5.7)
British Columbia	1 (2.9)
National ^a^	2 (5.7)
Other ^b^	2 (5.7)
Gender	
Reported	27 (77.1)
Not Reported	8 (22.9)
Data Collection	
Interviews	12 (34.3)
Survey/Questionnaire	11 (31.4)
Focus Groups	8 (22.9)
Other	7 (20.0)
Theory	
Theoretical	15 (48.9)
Atheoretical	20 (57.1)

Note. ^a^ National studies were categorized as studies that include greater than or equal to half of the Canadian Provinces and Territories or designated their scope as national

^b^ Other studies are multi-province/territory studies that do not exceed half of the Canadian Provinces or Territories but exceed one

Gender/Sex was reported in most studies (*n* = 27) (Antwi-Boasiako et al., 2022a, b; Archie et al., 2010; Bailey et al., 2024; Beausoleil et al., 2017; Boatswain-Kyte et al., 2024; Cénat et al., 2023; Clarke, 2011, 2012; Creese, 2019; Desruisseaux et al., 2002; Ellis et al., 2015; Hayle et al., 2016; Huculak & Peckmann, 2012; King et al., 2017; Knight et al., 2022; Litchmore, 2022; Manzo & Bailey, 2005; Mason et al., 2022; Millar, 2010; Ngobi et al., 2020; Noorishad et al., 2023; Osman et al., 2024; Owusu-Bempah et al., 2023; Ruck & Wortley, 2002; Syed et al., 2018; Waldron et al., 2023). Gender/Sex was predominantly reported as a dichotomous variable (*n* = 25Antwi-Boasiako et al., 2022a, b; Archie et al., 2010; Beausoleil et al., 2017; Boatswain-Kyte et al., 2024; Cénat et al., 2023; Clarke, 2011, 2012; Creese, 2019; Desruisseaux et al., 2002; Ellis et al., 2015; Hayle et al., 2016; Huculak & Peckmann, 2012; King et al., 2017; Knight et al., 2022; Manzo & Bailey, 2005; Mason et al., 2022; Millar, 2010; Ngobi et al., 2020; Noorishad et al., 2023; Osman et al., 2024; Owusu-Bempah et al., 2023; Ruck & Wortley, 2002; Syed et al., 2018; Waldron et al., 2023), with few studies focusing solely on a single gender/sex (*n* = 2) (Bailey et al., 2024; Litchmore, 2022).

Interviews (*n* = 12) (Adjei et al., 2018; Adjei & Minka, 2018; Alaazi et al., 2018; Clarke, 2011, 2012; Creese, 2019; Khalil et al., 2021; Litchmore, 2022; Manzo & Bailey, 2005; Mason et al., 2022; Ngobi et al., 2020; Osman et al., 2024) and survey/questionnaires (*n* = 11)(Antwi-Boasiako et al., 2021; Archie et al., 2010; Bailey et al., 2024; Beausoleil et al., 2017; Desruisseaux et al., 2002; Ellis et al., 2015; Hayle et al., 2016; King et al., 2017; Ruck & Wortley, 2002; Sibblis et al., 2022; Wortley & Tanner, 2005) were common data collection methods. Included studies were largely atheoretical or did not report a theory (*n* = 20) (Antwi-Boasiako et al., 2021, 2022a, b; Archie et al., 2010; Bailey et al., 2024; Beausoleil et al., 2017; Cénat et al., 2023; Clarke, 2011; Ellis et al., 2015; Huculak & Peckmann, 2012; Khalil et al., 2021; King et al., 2017; Knight et al., 2022; Mason et al., 2022; Millar, 2010; Owusu-Bempah et al., 2023; Ruck & Wortley, 2002; Syed et al., 2018; Waldron et al., 2023; Wortley & Tanner, 2005). Sample sizes ranged from a minimum sample size of 3 (Litchmore, 2022) to a maximum sample size of 16,177 (Owusu-Bempah et al., 2023).

**Fig. 2:**
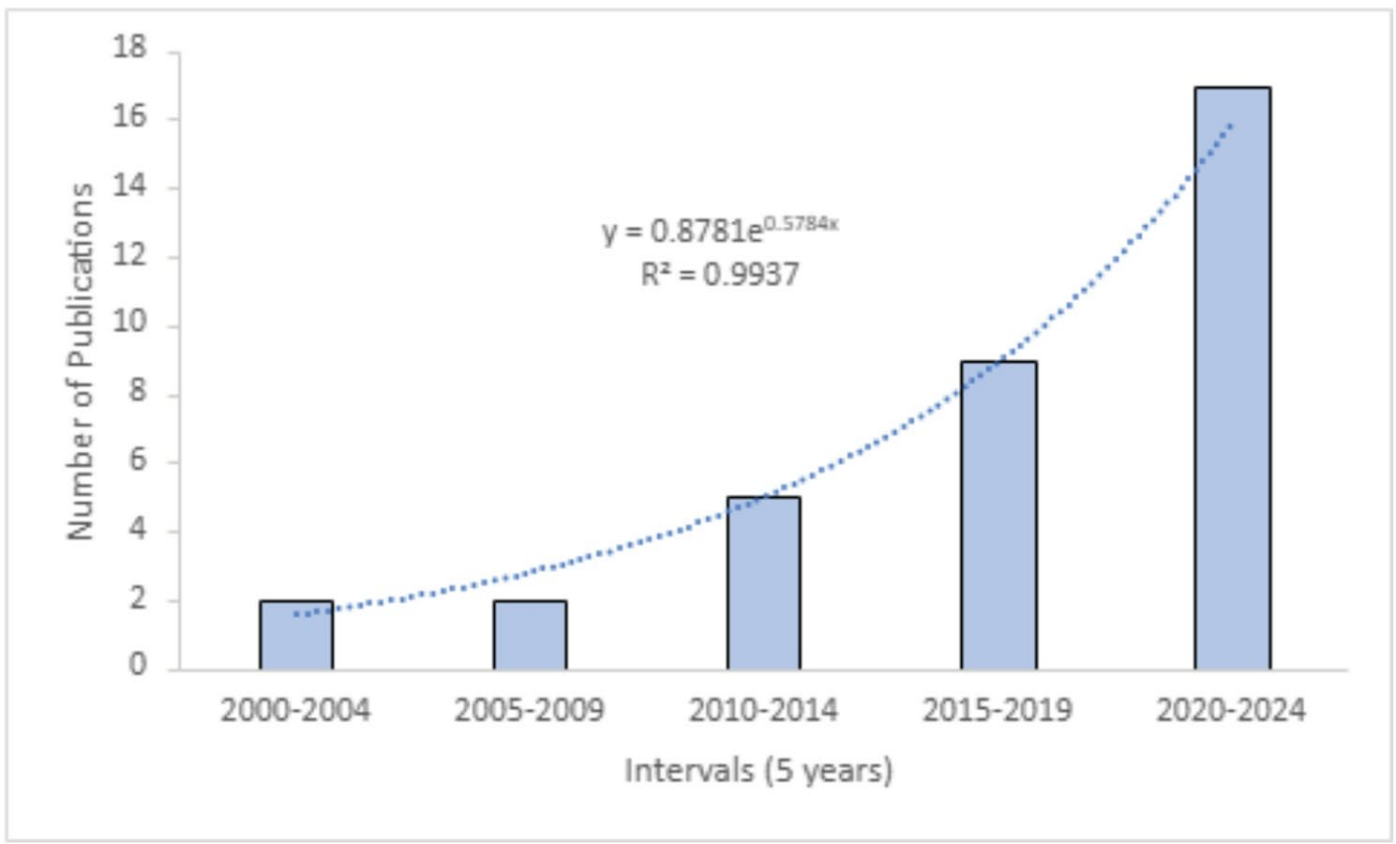
Publication Growth from 2000 to 2024 in 5-year intervals

No trends were indicated across individual years of publication; however, when grouped in five-year increments from the year 2000 to 2024, a strong exponential growth in publications on the topic was noted (see Fig. 2). Given a dearth of literature on this topic in a Canadian context, this growth is an important finding of this scoping review.

Studies explore a wide variety of age categories. Minimal consistency was noted in how age was reported across studies. Importantly, most studies did report an age category for participants (*n* = 25)(Alaazi et al., 2018; Archie et al., 2010; Bailey et al., 2024; Beausoleil et al., 2017; Boatswain-Kyte et al., 2024; Clarke, 2011, 2012; Creese, 2019; Desruisseaux et al., 2002; Ellis et al., 2015; Hayle et al., 2016; Huculak & Peckmann, 2012; King et al., 2017; Knight et al., 2022; Litchmore, 2022; Manzo & Bailey, 2005; Mason et al., 2022; Ngobi et al., 2020; Osman et al., 2024; Owusu-Bempah et al., 2023; Ruck & Wortley, 2002; Sibblis et al., 2022; Syed et al., 2018; Waldron et al., 2023; Wortley & Tanner, 2005). When breaking youth down into a dichotomized variable (children under 13 and youth up to age 30), few studies included children under 13 (*n* = 7) (Alaazi et al., 2018; Boatswain-Kyte et al., 2024; Huculak & Peckmann, 2012; King et al., 2017; Knight et al., 2022; Mason et al., 2022; Sibblis et al., 2022). All of the studies that included children under 13 overlapped with youth up to age 30 (e.g., Mason et al. (2022) reported on ages 12 to 18 years).

Participants’ country of origin was infrequently reported, with over half of the studies not including this characteristic (*n* = 21) (Antwi-Boasiako et al., 2021, 2022a; Bailey et al., 2024; Beausoleil et al., 2017; Boatswain-Kyte et al., 2024; Cénat et al., 2023; Clarke, 2011; Edwards et al., 2023; Hayle et al., 2016; Huculak & Peckmann, 2012; Khalil et al., 2021; King et al., 2017; Knight et al., 2022; Litchmore, 2022; Millar, 2010; Noorishad et al., 2023; Osman et al., 2024; Owusu-Bempah et al., 2023; Ruck & Wortley, 2002; Waldron et al., 2023; Wortley & Tanner, 2005). Of the studies that did report country of origin, there was no standard formatting. For example, one study defines participants as having “Black ancestors [who] emigrated to Canada from the West Indies, mostly Jamaica, rather than from the United States; an additional respondent’s family had immigrated from Africa” (Manzo & Bailey, 2005, p. 294). Other strategies existed reporting as African, Caribbean, or Black (e.g., Syed et al., 2018), reporting as regions (e.g., East Africa, West Africa) (e.g., Ngobi et al., 2020), while others reported at the country-level (e.g., Adjei et al., 2018; Adjei & Minka, 2018; Alaazi et al., 2018; Ellis et al., 2015).

Studies that noted a theory or theoretical framework more often reported the use of more than 1 theory than the use of a single theory. Critical Race Theory (CRT) was the most reported theory used (*n* = 6) (Adjei et al., 2018; Adjei & Minka, 2018; Clarke, 2012; Creese, 2019; Edwards et al., 2023; Noorishad et al., 2023). CRT was more often used in conjunction with another theory (*n* = 4, e.g., Creese (2019) uses CRT with Intersectional Feminism) rather than a stand-alone theory. This may indicate a perceived shortcoming of the theory to address the heterogeneity of Black Canadian youths. Feminist Theories were also used by some included studies (*n* = 3) (Clarke, 2012; Creese, 2019; Litchmore, 2022). Examples of other theories used included Transnational Theory (Alaazi et al., 2018), Conflict Theory (Hayle et al., 2016), and Anti-Black Racism (Boatswain-Kyte et al., 2024; Edwards et al., 2023).

### Narrative results

Analysis of the included studies reveals the pervasive and inescapable impact of the criminal justice system on the lives of Black youth. Studies focused on a variety of Canadian systems or institutional contexts but still reported links that directly relate to the criminal justice system. For example, Waldron et al. (2023) explored psychosis and other mental health problems or challenges of African Nova Scotian youth, a topic primarily situated in the health system but found that fear of police involvement was a driving factor for avoiding help-seeking. We synthesized key findings across institutions that frequently overlapped with the criminal justice system including child welfare, health, and education. A full summary of key findings, including studies that did not overlap with these institutions is available in Additional File 1.

#### Unpacking the criminal justice system

Studies that are explicitly focused on examining aspects of the criminal justice system demonstrate minimal overlap in findings due to differences across research purposes but highlight key areas that require further research (*n* = 7). For example Huculak and Peckmann (2012) explored in vivo tissue facial measurements used in forensic facial reconstructions. Hayle et al. (2016) and Wortley and Tanner (2005) examined experiences of being stopped and searched in youth. Syed et al. (2018) examined youth attitudes toward law enforcement, Owusu-Bempah et al. (2023) researched opioid overdose in incarcerated individuals, and Beausoleil et al. (2017) explored a reintegration intervention. Irrespective of differences in purpose, all studies point to a need for countering colour-evasiveness in Canadian institutions and a need for specific services and supports for Black Canadians who interact with the criminal justice system whether it be as a victim/survivor or perpetrator. Specifically, Huculak and Peckmann (2012) found that when compared with African American and European American children, African Nova Scotians have thicker facial tissue depths in the forehead, nose, mouth, and chin. These data suggest a need for specific considerations when undertaking forensic facial reconstructions of African Nova Scotian children and youth. Further, Beausoleil et al. (2017) point to a supportive reintegration approach that demonstrates better efficacy and cost-effectiveness than usual reintegration services for African Canadian youths. Consequently, these findings suggest that existing approaches to reintegration do not adequately consider the needs of African Canadian youth. Increased cost-effectiveness is a particularly interesting finding as it suggests that the criminal justice system itself may benefit from adopting redemption reintegration services.

Other studies focused on the criminal justice system highlighted disparities faced by incarcerated Black Canadians. In an exploration of individuals who had experienced incarceration in Ontario Owusu-Bempah et al. (2023) found that incarcerated Black Canadians were at an extremely high risk of opioid toxicity death (0.207 per 100 person years) compared with persons in the Ontario population who did not experience incarceration. Furthermore, findings indicate that two-weeks following post release was a particularly vulnerable time for opioid toxicity death. Further investigation is warranted into interventions that support individuals from the point of release to successful community reintegration. Two included studies noted that Black youth reported higher rates of being stopped and searched by police officers. Wortley and Tanner (2005) found in a large, random sample that students who self-identified as Black were more likely to report being stopped and searched by the police than students from any other racial background. A decade later, Hayle et al. (2016) found that racial differences in stop-and-search statistics existed only in a high school student sample when compared to a street youth sample. That is, race does not seem to influence the rate of police contact among street youth. However, within the student sample, Black students report higher rates of stop-and-search statistics compared to both other minority and white students. The differences between Black students and other racialized and/or minority students indicate the problematic façade created by the terminology visible minority, insofar as it masks experiences of Black populations as other racialized groups may experience better outcomes (Wortley & Tanner, 2005).

Two studies provided a unique perspective on engagement with the criminal justice system from the perspective of youth (Sibblis et al., 2022; Syed et al., 2018). Findings from these studies were categorized into the following themes: law enforcement has a positive effect on society, police institutions lack diversity, and officers sometimes abuse their powers. Black youth suggested that society problematically perceives police as a homogenous group and attribute negative interactions with police to the non-law enforcement individual involved in the situation (Syed et al., 2018). These opinions were complex and existed alongside discussions of negative experiences with law enforcement (Syed et al., 2018). Youth also perceived a lack of diversity in the police force resulting in white Eurocentric thinking and stereotyping by officers, and that officers sometimes abuse their powers (e.g., racial profiling, obstructing justice, being overly aggressive) (Syed et al., 2018). Indeed, Sibblis et al. (2022) found that Black people who immigrated to Canada as children and grew up within Canadian institutions and systems (the 1.5 generation) are more likely to distrust police, the criminal justice system, and Canadian institutions more broadly. These findings indicate the existence of complex opinions and experiences with law enforcement and the criminal justice system among Black youth in Canada. They support the notion of heterogeneity of the Black population in Canada.

#### Child welfare: a pipeline to criminal justice involvement

Overlap between the criminal justice system and child welfare system was prominent among included studies (*n* = 14). Findings from these studies indicate disparate outcomes for Black families and children involved with these systems. Boatswain-Kyte et al. (2024) found that Black children were more likely to crossover from the child welfare system to youth justice, and Black children were almost twice as likely to be sentenced to detention than white children in Quebec. Further, Black men/fathers are overrepresented among those in prison for support-debt-related charges (Millar, 2010). Black families were also found to perceive differential treatment within child welfare services that related to criminalization and the criminal justice system. Specifically, mothers perceived that they were often criminalized through their involvement with the child welfare system (Clarke, 2011, 2012). This is evidenced by the discussions of increased assault charges due to increased police involvement in investigations (Clarke, 2012). Further, youths felt they experienced hyper surveillance and differential treatment from police (Clarke, 2011, 2012).

Differential treatment of children and families of colour within and across systems is well documented (Cénat et al., 2021; Derezotes et al., 2005; Dettlaff & Boyd, 2021). Ontario’s mandatory reporting requirement (Duty to Report) was noted as connecting the criminal justice system and the child welfare systems through varying mechanisms. Mandatory reporters such as the police, schools, and healthcare professionals were discussed by child welfare workers and community staff as holding biases that influence their likelihood to report Black families to the child welfare system (Antwi-Boasiako et al., 2022a, b). The mandatory reporting requirement and Canadian laws were found to be particularly difficult for newcomer families to adjust to (Noorishad et al., 2023). The absence of specific language in relation to Black families within Ontario’s *Child and Family Services Act* may be a potential contributor to biases as it enacts and promotes a ‘colour-evasive’ lens toward child welfare within the province (Edwards et al., 2023).

Collectively, the findings demonstrate potential processes that link the child welfare system to the criminal justice system. Findings around Canadian newcomers and cultural differences in parenting indicate that cultural differences in disciplinary styles may put newcomer families at a higher risk of child welfare involvement (Cénat et al., 2023). Consequently, child welfare involvement results in children being separated from their families for manifestations of anti-Black racism such as misunderstandings by mandatory reporters on the nutritional value of cultural foods (Cénat et al., 2023) or around cultural language-use (Adjei & Minka, 2018). Following this separation, Black youth experience higher crossover into youth justice (Boatswain-Kyte et al., 2024). This may in part be to experiences of anti-Black racism within foster homes (Clarke, 2012). At each step along this process, legislation enables these actions through its colour-evasive lens (Edwards et al., 2023). These processes linking the child welfare and criminal justice systems in Canada require further attention and research to disentangle the manifestations of anti-Black racism in the form of disproportionate representation in the child welfare system and criminal justice system and move toward developing targeted interventions for reducing these disparities.

#### Health services: fear, avoidance and coercion

Studies that focused on health or healthcare topics suggest that health system or healthcare avoidance is linked to the criminal justice system’s permeation into every aspect of the lives of Black youths. Studies indicated that a fear of police involvement in healthcare or health matters prevents help-seeking and screening behaviours (Ngobi et al., 2020; Waldron et al., 2023). Waldron et al. (2023) found that African Nova Scotian caregivers perceived healthcare avoidance as a product of a fear of police involvement in care and that community leaders believed that mentally ill individuals were more commonly addressed through negative police interactions rather than mental health services. Additionally, the criminalization of HIV non-disclosure was found to be a significant barrier to testing for young heterosexual African migrants in Ottawa (Ngobi et al., 2020). Another study exploring HIV risk among youth highlighted a belief among community leaders that the social environment, specifically the lack of community programs, is a mechanism that leads youth into police involvement, unsafe sexual activities, and drug use (Khalil et al., 2021).

The connection to the criminal justice system persisted even within healthcare settings. Knight et al. (2022) found that Black patients experiencing first-episode psychosis (FEP) were more likely to be coercively referred to FEP programs by police, ambulance services, or court orders compared to other patients. They were also more likely to undergo coercive interventions, both medical and legal, with a higher likelihood of facing legal coercion specifically. Archie et al. (2010) reported that 22.7% of help-seeking among Black participants was initiated by police involvement; however, this rate did not significantly differ from that of other ethnic groups, including white, Asian, and other ethnicities. Experiences of anti-Black racism that discourage the use of health services may lead Black youth to seek care later in the progression of a disease or illness, increasing the likelihood of prior interactions with the criminal justice system.

#### Education system: all eyes on you

The crossover between the education system and the criminal justice system was evidenced in included studies. Specifically, police presence in the school setting was discussed. Two of the included studies discussed experiences of anti-Black racism in the school setting (Mason et al., 2022; Ruck & Wortley, 2002). Mason et al. (2022) findings indicate hyper surveillance of Black youth within school by staff and police officer. Experiences of hyper surveillance tied in broadly to experiences of anti-Black racism within the school setting that also included negative stereotypes about Black students and being assigned to courses that do not allow for progression into postsecondary education. Ruck and Wortley’s (2002) findings concur, indicating that Black students were more likely to believe that students from their racial group were more likely to have the police called on them and to believe that they would be treated worse or much worse by police at school.

One study found that Black Female youths invoked “rape myths” with respect to sexual violence in the school setting (Litchmore, 2022). That is, learned “ideologies that excuse sexual violence against women and advocate that women should accept responsibility for their sexual victimization” (Deming et al., 2013, p. 467). Participants’ discussions positioned the victim’s personality as ‘sexually promiscuous’ and as someone “who invited responses from the young men whom she was involved” (Litchmore, 2022, p. 243). Litchmore (2022) concludes that such discourses may be interpreted as indicative of an oppressive school environment where young women inadvertently invoked discourses that marginalized themselves. These findings support the claim for a proactive gender-based violence policies within the school setting (Litchmore, 2022).

## Discussion

This study conducted a scoping review of Black youth involvement in the Canadian criminal justice system to explore the state of the literature and identify knowledge gaps. In doing so, this study contributed to addressing a significant gap in the literature, as research on this Black populations within the Canadian context remains limited (Etowa et al., 2007). Even more concerning is the lack of disaggregated race-based data which is essential for a deeper understanding of these issues (Moscher & Mahon-Haft, 2010; Reasons et al., 2016). Our scoping review included 35 studies, aligning with the notion of a lack of literature. This underscores the need for further research to improve understanding and advance knowledge on Black youth experiences with the Canadian criminal justice system.

Our narrative synthesis categorized findings across Canadian institutions. Findings pertaining primarily to the criminal justice system highlight that Black Canadian youth disproportionately experience police stops and searches (Hayle et al., 2016; Wortley & Tanner, 2005), a pattern commonly observed among racialized youth (Flacks, 2018; Quinton, 2024). In response to these trends, Quinton (2024) proposes a theoretical model aimed at enhancing the understanding of the socio-ecological factors that influence stop-and-search decisions and shape these interactions. The findings also indicate a risk of opioid toxicity mortality, particularly within the first two weeks following release from incarceration (Owusu-Bempah et al., 2023). Previous studies have demonstrated the positive effects of opioid agonist treatment (OAT) in mitigating these risks (Chaillon et al., 2022; Russel et al., 2023). Specifically, Chaillon et al. (2022) reported that prison-based OAT programs prevented 1.8 deaths per 100 person-years during the first-year post-incarceration and 3.4 deaths per 100 person-years within the first month post-release. Further research is needed to deepen the understanding of opioid use among Black Canadian youth and to develop effective support strategies, particularly during the two weeks following release.

Collectively, these findings highlight a deeply rooted interconnection between Canada’s child welfare system and the criminal justice system. The two systems share a synergistic relationship, where involvement in one often increases the likelihood of interaction with the other. The child welfare system has often been described as a pipeline funneling children into the criminal justice system (Mohamud et al., 2021). Research has highlighted the criminalization of Black youth within Ontario’s child welfare system (Gharabhagi et al., 2016), showing that Black youth are underrepresented in mental health services while being overrepresented in facilities focused on containment and control. Furthermore, mandatory reporting laws may function as tools for policing the behaviors of Black parents (Raz, 2020). Despite this, little research on mandatory reporting laws and their impact on Black youth and families was identified outside of Ontario. To disrupt the flow of Black youth from the child welfare system into the criminal justice system, comprehensive, multi-level interventions are needed to address policy shortcomings and challenge systemic biases among child welfare professionals.

The intersection of the criminal justice system and mental health care services has been well-documented in scholarly literature (Domino et al., 2019; Heyman, 2020; Hon. Mr. Justice Richard D. Schneider, 2015; Livingston, 2016). Our finding of low healthcare utilization among Black populations, particularly regarding mental health services, aligns with previous research (Fenta et al., 2006; Fields-Oriogun et al., 2024; Whitley et al., 2017). This low utilization can lead to the worsening of mental health conditions, which may result in behaviors perceived as threatening, thereby increasing the likelihood of police involvement. Conversely, the criminalization of Black individuals with mental health disorders has been identified as a significant barrier to accessing health services (Anucha et al., 2017; Bala et al., 2013). Schneider (2015) describes the ‘forensic patient’ as a byproduct of systemic failures within the healthcare system to adequately support individuals with mental health disorders, leading to condition escalation and eventual law enforcement involvement—often triggered by family members calling the police after premature discharge from healthcare facilities. This pathway may help explain the high rates of coercive interactions with the healthcare system observed among Black youth in the studies reviewed. Future research is needed to explicate the pathways between health service utilization and criminal justice involvement for Black youth in Canada and interventions should focus on improving early utilization of health services.

The permeation of the criminal justice system into the schooling of experiences of Black Youth is well-evidenced and documented (Barnes & Motz, 2018; Jenkins & Warren, 2024). Included studies reported higher rates of surveillance experienced by Black youth in the school context (Mason et al., 2022; Ruck & Wortley, 2002). Hyper-surveillance may result in higher rates of school discipline (e.g., suspension or expulsion), an entry point into the STPP (Barnes & Motz, 2018). While the STPP extends across the entire school setting, teachers play an important role as the primary point of contact for students. In addressing the STTP, Bryan (2017) outlines six avenues for pre-service teacher education including training in culturally relevant pedagogy and the establishment of Afrocentric schools. Nance (2016) also recommends teacher training to address the STPP, suggesting behavioural problems are correlated to a teachers’ ability to manage the classroom and engage students. Focusing on pre-service teacher training may be one mechanism for reducing biases that lead to hyper surveillance and over reporting of Black students in the school setting.

### Strengths and limitations

To the best of our knowledge, this is the first study in Canada to synthesize existing literature on Black youth and their interactions with the Canadian criminal justice system. Our findings contribute to the current body of research by providing clearer insights and advancing understanding of this critical issue. This study will engage governments, policymakers, and stakeholders in developing and implementing effective solutions to address the persistent and serious problem of Black youth overrepresentation in the justice system. Despite efforts over the past decade, significant disparities in incarceration rates between Black and white youth persist. By highlighting these injustices, our research encourages stakeholders to critically examine and dismantle racial policies and practices that perpetuate systemic racism. Additionally, the findings reveal several gaps in existing knowledge, indicating the need for further research to explore the underlying causes, consequences, and potential solutions to this issue. Addressing the overrepresentation of Black youth in the Canadian criminal justice system requires a thorough understanding of its root causes and contributing factors.

While this scoping review has notable strengths, it also presents some limitations. The broad approach to reviewing literature on Black youth, followed by the extraction of studies specific to the criminal justice system, may have limited the scope of the research. Additionally, within the criminal justice system, individuals aged 18 and older are classified as adults. As a result, our initial search focusing on “youth” may have overlooked studies examining offenders within our target age range (0–30 years) due to this variation in terminology. Future research on the criminal justice system and youth should account for these age classification differences to ensure comprehensive coverage Further, a critical appraisal of the literature may strengthen our findings and provide clear direction for improving the available data. Nevertheless, this scoping review establishes a foundation for ongoing research on Black youth and the criminal justice system to ensure that evidence-based findings drive meaningful actions to improve the lives of Black youth.

## Conclusions

Black youth continue to be overrepresented in the Canadian criminal justice system. Despite the severity of this issue, scholarly research on the topic in Canada remains limited. This study conducted a scoping review of the existing literature on Black youth and their interactions with the Canadian criminal justice system. The findings highlight the disproportionate representation of Black youth within the system, leading to serious health and social consequences. Notably, the child welfare system often serves as a pipeline to the criminal justice system for Black youth in Canada. While certain policies and programs aim to address this issue, much more work is needed to effectively reduce or prevent Black youth involvement with the justice system. The Canadian government, policymakers, and stakeholders must intensify their efforts and initiatives to mitigate this problem and improve the mental health and social outcomes of Black Canadian youth. Additionally, the study reveals significant research and knowledge gaps. Although some studies have examined Black youth and the Canadian criminal justice system, they only begin to address the depth of the issue. Beneath the surface, there are complex factors that require research and policy attention. Therefore, further empirical research is necessary to gain a comprehensive understanding of this problem and to develop effective solutions. In particular, more research is needed to explore the connection between the child welfare system and the criminal justice system, and how this pathway can be disrupted.

## Supplementary Information

Supplementary Material 1.

Supplementary Material 2.

## Data Availability

No datasets were generated or analysed during the current study.

## Abbreviations

CRT: Critical Race Theory
FEP: First–Episode Psychosis
OAT: Opioid Agonist Treatment
PRISMA: ScR–Reporting Items for Systematic Reviews and Meta–Analyses extension for Scoping Reviews
STPP: School to prison pipeline

## References

[CR1] Adjei, P. B., & Minka, E. (2018). Black parents ask for a second look: Parenting under ‘White’ Child Protection rules in Canada. *Children and Youth Services Review,**94*, 511–524. 10.1016/j.childyouth.2018.08.030

[CR2] Adjei, P. B., Mullings, D., Baffoe, M., Quaicoe, L., Abdul-Rahman, L., Shears, V., & Fitzgerald, S. (2018). The “Fragility of Goodness”: Black parents’ perspective about raising children in Toronto, Winnipeg, and St. John’s of Canada. *Journal of Public Child Welfare,**12*(4), 461–491. 10.1080/15548732.2017.1401575

[CR3] Alaazi, D. A., Salami, B., Yohani, S., Vallianatos, H., Okeke-Ihejirika, P., & Nsaliwa, C. (2018). Transnationalism, parenting, and child disciplinary practices of African immigrants in Alberta, Canada. *Child Abuse & Neglect,**86*, 147–157. 10.1016/j.chiabu.2018.09.013 30292095 10.1016/j.chiabu.2018.09.013

[CR4] Alhassan, J. A. K., Khare, N. S., & Tanvir, A. (2024). Variegated racism: Exploring experiences of anti-Black racism and their progression in medical education. *CMAJ,**196*(22), E751–E759. 10.1503/cmaj.231753 38857932 10.1503/cmaj.231753 PMC11173657

[CR5] Antwi-Boasiako, K., Fallon, B., King, B., Trocmé, N., & Fluke, J. (2021). Examining decision-making tools and child welfare involvement among Black families in Ontario, Canada. *Children and Youth Services Review,**126*, Article 106048. 10.1016/j.childyouth.2021.106048

[CR6] Antwi-Boasiako, K., Fallon, B., King, B., Trocmé, N., & Fluke, J. (2022a). Addressing the overrepresentation of Black children in Ontario’s child welfare system: Insights from child welfare workers and community service providers. *Child Abuse & Neglect,**123*, Article 105423. 10.1016/j.chiabu.2021.105423 34871923 10.1016/j.chiabu.2021.105423

[CR7] Antwi-Boasiako, K., Fallon, B., King, B., Trocmé, N., & Fluke, J. (2022b). Understanding the overrepresentation of Black children in Ontario’s child welfare system: Perspectives from child welfare workers and community service providers. *Child Abuse & Neglect,**123*, Article 105425. 10.1016/j.chiabu.2021.105425 34890960 10.1016/j.chiabu.2021.105425

[CR8] Anucha, U., Srikanthan, S., Siad-Togane, R., & Galabuzi, G. E. (2017). *Doing Right Together for Black Youth: What We Learned from the Community Engagement Sessions for the Ontario Black Youth Action Plan - Youth Research and Evaluation eXchange*. YouthREX. https://youthrex.com/report/doing-right-together-for-black-youth-what-we-learned-from-the-community-engagement-sessions-for-the-ontario-black-youth-action-plan/, https://youthrex.com/report/doing-right-together-for-black-youth-what-we-learned-from-the-community-engagement-sessions-for-the-ontario-black-youth-action-plan/

[CR9] Archie, S., Akhtar-Danesh, N., Norman, R., Malla, A., Roy, P., & Zipursky, R. B. (2010). Ethnic diversity and pathways to care for a first episode of psychosis in Ontario. *Schizophrenia Bulletin,**36*(4), 688–701. 10.1093/schbul/sbn137 18987101 10.1093/schbul/sbn137 PMC2894595

[CR10] Arksey, H., & O’Malley, L. (2005). Scoping studies: Towards a methodological framework. *International Journal of Social Research Methodology*, *8*(1), 19–32. 10.1080/1364557032000119616

[CR11] Bailey, A., Bailey, R., Newman, G., Barrett, A., Nguyen, M., & Lindsay, J. (2024). Deconstructing the trauma-altered identity of black men. *Journal of Child & Adolescent Trauma,**17*(3), 999–1012. 10.1007/s40653-023-00526-0 39309334 10.1007/s40653-023-00526-0 PMC11413278

[CR12] Bala, N. C., De Filippis, R., & Hunter, K. (2013). *Crossover youth: Improving Ontario’s responses*. Ontario Chapter of the Association of Family & Concilliation Courts. https://afccontario.ca/wp-content/uploads/2015/11/Bala-De-Filippis-Hunter-Crossover-Kids.pdf

[CR13] Barnes, J. C., & Motz, R. T. (2018). Reducing racial inequalities in adulthood arrest by reducing inequalities in school discipline: Evidence from the school-to-prison pipeline. *Developmental Psychology,**54*(12), 2328–2340. 10.1037/dev0000613 30265031 10.1037/dev0000613

[CR14] Beausoleil, V., Renner, C., Dunn, J., Hinnewaah, P., Morris, K., Hamilton, A., Braithewaite, S., Hunter, N., Browne, G., & Browne, D. T. (2017). The effect and expense of redemption reintegration services versus usual reintegration care for young African Canadians discharged from incarceration. *Health & Social Care in the Community*, *25*(2), 590–601. 10.1111/hsc.12346 27038240 10.1111/hsc.12346

[CR15] Bernard, W., & Smith, H. (2018). Injustice, justice, and Africentric practice in Canada. *Canadian Social Work Review = Revue Canadienne De Service Social,**35*(1), 149–157. 10.7202/1051108ar

[CR16] Boateng, G., & Regragui, S. (2023, April 13). *Project Launch of Retooling Black Anxiety: Examining Anxiety and Mental Health Issues Among Black Youth*. Dahdaleh Institute for Global Health Research. https://www.yorku.ca/dighr/project-launch-of-retooling-black-anxiety/

[CR17] Boatswain-Kyte, A., Hélie, S., & Royer, M.-N. (2024). A critical examination of youth service trajectories: Black children’s transition from child welfare to youth justice. *Children and Youth Services Review,**157*, Article 107411. 10.1016/j.childyouth.2023.107411

[CR18] Bryan, N. (2017). White teachers’ role in sustaining the school-to-prison pipeline: Recommendations for teacher education. *The Urban Review,**49*(2), 326–345. 10.1007/s11256-017-0403-3

[CR19] Cénat, J. M., McIntee, S.-E., Mukunzi, J. N., & Noorishad, P.-G. (2021). Overrepresentation of black children in the child welfare system: A systematic review to understand and better act. *Children and Youth Services Review,**120*, Article 105714. 10.1016/j.childyouth.2020.105714

[CR20] Cénat, J. M., Noorishad, P.-G., Czechowski, K., Mukunzi, J. N., Hajizadeh, S., McIntee, S.-E., & Dalexis, R. D. (2023). The seven reasons why Black children are overrepresented in the Child Welfare System in Ontario (Canada): A qualitative study from the perspectives of caseworkers and community facilitators. *Child & Adolescent Social Work Journal,**40*(5), 655–670. 10.1007/s10560-021-00793-6

[CR21] Chaillon, A., Bharat, C., Stone, J., Jones, N., Degenhardt, L., Larney, S., Farrell, M., Vickerman, P., Hickman, M., Martin, N. K., & Bórquez, A. (2022). Modeling the population-level impact of opioid agonist treatment on mortality among people accessing treatment between 2001 and 2020 in New South Wales, Australia. *Addiction,**117*(5), 1338–1352. 10.1111/add.15736 34729841 10.1111/add.15736 PMC9299987

[CR22] Clarke, J. (2011). The challenges of child welfare involvement for Afro-Caribbean families in Toronto. *Children and Youth Services Review*, *33*(2), 274–283. 10.1016/j.childyouth.2010.09.010

[CR23] Clarke, J. (2012). Beyond child protection: Afro-Caribbean service users of child welfare. *Journal of Progressive Human Services*, *23*(3), 223–257. 10.1080/10428232.2012.719119

[CR24] Cotter, A. (2022). Experiences of discrimination among the Black and Indigenous populations in Canada, 2019. *Juristat: Canadian Centre for Justice Statistics*, 1–14.

[CR25] Creese, G. (2019). Growing up African Canadian in Vancouver: Racialization, gender and sexuality. *Canadian Journal of Sociology,**44*(4), 425–446. 10.29173/cjs29456

[CR26] Deming, M. E., Covan, E. K., Swan, S. C., & Billings, D. L. (2013). Exploring rape myths, gendered norms, group processing, and the social context of rape among college women: A qualitative analysis. *Violence Against Women,**19*(4), 465–485. 10.1177/1077801213487044 23637315 10.1177/1077801213487044

[CR27] Department of Justice (2024). *State of the Criminal Justice System Dashboard*. Government of Canada. https://www.justice.gc.ca/socjs-esjp/en/dash-tab/lm-sp

[CR28] Department of Justice Government of Canada (2022, April 7). *Black Youth and the Criminal Justice System: Summary Report of an Engagement Process in Canada*. https://www.justice.gc.ca/eng/rp-pr/jr/bycjs-yncjs/background-contexte.html

[CR29] Department of Justice Government of Canada (2014, January 17). Chapter 1 *- Background - Making the Links in Family Violence Cases: Collaboration among the Family, Child Protection and Criminal Justice Systems*. Government of Canada. https://www.justice.gc.ca/eng/rp-pr/cj-jp/fv-vf/mlfvc-elcvf/p3.html

[CR30] Department of Canadian Heritage (2024, October 1). *Changing Systems, Transforming Lives: Canada’s Anti-Racism Strategy 2024–2028—Canada.ca*. Government of Canada. https://publications.gc.ca/collections/collection_2025/pch/CH37-4-29-2024-eng.pdf

[CR31] Derezotes, D. M., Poertner, J., & Testa, M. F. (2005). *Race Matters in Child Welfare: The Overrepresentation of African American Children in the System*. Child Welfare League of America.

[CR32] Desruisseaux, J.-C., St-Pierre, L., Tougas, F., & de la Sablonnière, R. (2002). Jeunes Haïtiens de Montréal et Déviance: Frustration, méfiance et mauvaises fréquentations. [Young Haitians in Montreal and deviance: Frustration, mistrust, and bad company.]. *Revue Québécoise De Psychologie,**23*(3), 43–55.

[CR33] Dettlaff, A. J., & Boyd, R. (2021). Racial disproportionality and disparities in the Child Welfare System: Why do they exist, and what can be done to address them? - Alan J. Dettlaff, Reiko Boyd, 2020. *The ANNALS of American Academy of Political and Social Sciences,**692*(1), 253–274. 10.1177/0002716220980329

[CR34] Domino, M. E., Gertner, A., Grabert, B., Cuddeback, G. S., Childers, T., & Morrissey, J. P. (2019). Do timely mental health services reduce re-incarceration among prison releasees with severe mental illness? *Health Services Research*, *54*(3), 592–602. 10.1111/1475-6773.13128 30829406 10.1111/1475-6773.13128 PMC6505414

[CR35] Dryden, O., & Nnorom, O. (2021). Time to dismantle systemic anti-Black racism in medicine in Canada. *Canadian Medical Association Journal*, *193*(2), E55–E57. 10.1503/cmaj.201579 33431548 10.1503/cmaj.201579 PMC7773037

[CR36] Edwards, T., McManamna, N., & King, B. (2023). The absence of language: A critical race discourse analysis of ontario’s child welfare legislation and the impacts on black families. *Child Abuse & Neglect*, *143*, 106249. 10.1016/j.chiabu.2023.106249 37290205 10.1016/j.chiabu.2023.106249

[CR37] Ellis, B. H., Abdi, S. M., Miller, A. B., White, M. T., & Lincoln, A. K. (2015). Protective factors for violence perpetration in Somali young adults: The role of community belonging and neighborhood cohesion. *Psychology of Violence,**5*(4), 384–392. 10.1037/a0039610

[CR38] Etowa, J. B., Wiens, J., Bernard, W. T., & Clow, B. (2007). Determinants of Black women’s health in rural and remote communities. *Canadian Journal of Nursing Research = Revue Canadienne de Recherche En Sciences Infirmieres,**39*(3), 56–76. 17970460

[CR39] Fenta, H., Hyman, I., & Noh, S. (2006). Mental health service utilization by Ethiopian immigrants and refugees in Toronto. *Journal of Nervous and Mental Disease,**194*(12), 925–934. 10.1097/01.nmd.0000249109.71776.58 17164631 10.1097/01.nmd.0000249109.71776.58

[CR40] Fields-Oriogun, D., Foley-Nicpon, M., & Thornburg-Suresh, M. (2024). Mental health stigma and service use among black American youth: A systematic review. *American Journal of Orthopsychiatry*, *94*(6), 655–667. 10.1037/ort0000749 38934907 10.1037/ort0000749

[CR41] Flacks, S. (2018). The stop and search of minors: A vital police tool? *Criminology & Criminal Justice,**18*(3), 364–384.

[CR42] Gharabhagi, K., Trocmé, N., & Newman, D. (2016). *Because Young People Matter: REport of the Residential Services Review Panel*. Ministry of Children and Youth Services.

[CR43] Government of Canada Office of the Correctional Investigator (2013). *A Case Study of Diversity in Corrections: The Black Inmate Experience in Federal Penitentiaries* (p. 33). The Office of the Correctional Investigator. https://oci-bec.gc.ca/sites/default/files/2024-04/oth-aut20131126-eng.pdf

[CR44] Hayle, S., Wortley, S., & Tanner, J. (2016). Race, street life, and policing: Implications for racial profiling. *Canadian Journal of Criminology & Criminal Justice,**58*(3), 322–353. 10.3138/cjccj.2014.E32

[CR45] Heyman, I. (2020). *People in mental distress, police and out-of-hours health services: A qualitative exploratory case study of experiences and the intersect of safeguarding services.* https://rgu-repository.worktribe.com/output/1357998

[CR46] Schneider, Richard D.. (2015). *The Mentally Ill: How They Became Enmeshed in the Criminal Justice System and How We Might Get Them Out*. Department of Justice Canada.

[CR47] Huculak, M. A., & Peckmann, T. R. (2012). In vivo facial tissue depth study of African Nova Scotian children. *Canadian Society of Forensic Science Journal*, *45*(3), 126–142. 10.1080/00085030.2012.10757186

[CR48] Husbands, W., Lawson, D. O., Etowa, E. B., Mbuagbaw, L., Baidoobonso, S., Tharao, W., Yaya, S., Nelson, L. E., Aden, M., & Etowa, J. (2022). Black Canadians’ exposure to everyday racism: Implications for health system access and health promotion among Urban Black Communities. *Journal of Urban Health,**99*(5), 829–841. 10.1007/s11524-022-00676-w 36066788 10.1007/s11524-022-00676-w PMC9447939

[CR49] Jenkins, D. A., & Warren, C. A. (2024). Towards anti-carceral leadership: Remaking public schools to refuse Black students’ surveillance, containment, and control. *Educational Policy,**38*(3), 624–641. 10.1177/08959048231220024

[CR50] Jennifer White (2021, January). *Working with Specific Groups of Children and Youth at Risk of Suicide*. Ministry of Children and Family Development. https://www2.gov.bc.ca/assets/gov/health/managing-your-health/mental-health-substance-use/child-teen-mental-health/guide_for_working_with_specific_groups_of_children_and_youth_at_risk_for_suicide.pdf

[CR51] Khalil, C., Goldfarb, R., Travers, R., Coleman, T., ACBY Study Team. (2021). HIV risk in African, Caribbean and Black youth: Perceptions of community leaders in the Windsor-Sussex Region. - Dalhousie University. *University of Toronto Medical Journal,**98*(3), 38–44.

[CR52] King, B., Fallon, B., Boyd, R., Black, T., Antwi-Boasiako, K., & O’Connor, C. (2017). Factors associated with racial differences in child welfare investigative decision-making in Ontario, Canada. *Child Abuse & Neglect,**73*, 89–105. 10.1016/j.chiabu.2017.09.027 28950215 10.1016/j.chiabu.2017.09.027

[CR53] Knight, S., Jarvis, G. E., Ryder, A. G., Lashley, M., & Rousseau, C. (2022). Ethnoracial differences in coercive referral and intervention among patients with first-episode psychosis. *Psychiatric Services (Washington, D. C.),**73*(1), 2–8. 10.1176/appi.ps.202000715 34253035 10.1176/appi.ps.202000715

[CR54] Litchmore, R. V. (2022). She’s very known in the school”: Black girls, race, gender, and sexual violence in Ontario schools. *Qualitative Psychology,**9*(3), 232–250. 10.1037/qup0000221

[CR55] Livingston, J. D. (2016). Contact between police and people with mental disorders: A review of rates. *Psychiatric Services,**67*(8), 850–857. 10.1176/appi.ps.201500312 27079990 10.1176/appi.ps.201500312

[CR56] Manzo, J. F., & Bailey, M. M. (2005). On the assimilation of racial stereotypes among Black Canadian young offenders*. *Canadian Review of Sociology/Revue Canadienne de Sociologie,**42*(3), 283–300. 10.1111/j.1755-618X.2005.tb00841.x

[CR57] Mason, A., Salami, B., Fouché, C., Richter, S., Sibeko, L., & Adekola, S. (2022). Aspirations, schooling experiences, and educational outcomes of African migrant children in Canada. *Canadian Ethnic Studies,**54*(2), 1–22. 10.1353/ces.2022.0013

[CR58] Maynard, R. (2017). *Policing black lives: State violence in Canada from slavery to the present*. Fernwood Publishing.

[CR59] Millar, P. (2010). Punishing our way out of poverty: The prosecution of child-support debt in Alberta, Canada. *Canadian Journal of Law and Society,**25*(2), 149–166.

[CR60] Mohamud, F., Edwards, T., Antwi-Boasiako, K., William, K., King, J., Igor, E., & King, B. (2021). Racial disparity in the Ontario child welfare system: Conceptualizing policies and practices that drive involvement for Black families. *Children and Youth Services Review,**120*, Article 105711. 10.1016/j.childyouth.2020.105711

[CR61] Moscher, C., & Mahon-Haft, T. (2010). Race, crime and criminal justice in Canada. In A. Kolunta-Crumpton (Ed.), *Race, crime and criminal justice: International perspectives* (p. 341). Palgrace Macmillan UK.

[CR62] Nance, J. P. (2016). Dismantling the school-to-prison pipeline: Tools for change. *Arizona State Law Journal,**48*, 313.

[CR63] Ngobi, J. B., Pottie, K., Leonard, L., Tugwell, P., Hoffman, S., & Welch, V. (2020). Multi-level barriers to reaching HIV testing among young heterosexual African migrants from HIV-endemic countries in Ottawa. *The Canadian Journal of Human Sexuality,**29*(1), 79–93. 10.3138/cjhs.2019-0033

[CR64] Noorishad, P.-G., Paul Darius, W., Czechowski, K., McIntee, S.-E., Ntunga Mukunzi, J., & Mary Cénat, J. (2023). Racism as a vehicle for the overrepresentation of Black youth in child protection services in Ontario, Canada: Caseworkers’ and community facilitators’ perspectives. *Children and Youth Services Review,**149*, Article 106963-. 10.1016/j.childyouth.2023.106963

[CR65] Ontario Human Rights Commission (2018). *Interrupted Childhoods: Over-representation of Indigenous and Black children in Ontario child welfare* (p. 73). Ontario Human Rights Commission. https://www3.ohrc.on.ca/sites/default/files/Interrupted%20childhoods_Over-representation%20of%20Indigenous%20and%20Black%20children%20in%20Ontario%20child%20welfare_accessible.pdf

[CR66] Osman, S., Aiello, O., Brouillette, K., Taylor, M., McKenzie, K., Renzaho, A. M. N., Henderson, J., Hamilton, H., & Salami, B. (2024). Dual pandemics”: Intersecting influences of anti-Black racism and the COVID-19 pandemic on the mental health of Black youth. *Canadian Journal of Nursing Research,* , Article 08445621241253116. 10.1177/08445621241253116 10.1177/08445621241253116 PMC11967086 38751058

[CR67] Owusu-Bempah, A., Nicholson, H. L., Butler, A., Croxford, R., & Kouyoumdjian, F. G. (2023). Opioid toxicity deaths in Black persons who experienced provincial incarceration in Ontario, Canada 2015–2020: A population-based study. *Preventive Medicine,**177*, Article 107778. 10.1016/j.ypmed.2023.107778 37967621 10.1016/j.ypmed.2023.107778

[CR68] Quinton, P. (2024). From estimating to explaining and eliminating ethnic disproportionality in stop and search. *The Political Quarterly*, *95*(3), 489–497. 10.1111/1467-923X.13433

[CR69] Raz, M. (2020). Calling child protectives services is a form of community policing that should be used appropriately: Time to engage mandatory reporters as to the harmful effects of unnecessary reports. *Children and Youth Services Review,**110*, Article 104817. 10.1016/j.childyouth.2020.104817

[CR70] Reasons, C., Bige, M., Paras, C., & Arora, S. (2016). Race and criminal justice in Canada. *International Journal of Criminal Justice Sciences*, *11*(2), 75.

[CR71] Ruck, M. D., & Wortley, S. (2002). Racial and ethnic minority high school students’ perceptions of school disciplinary practices: A look at some Canadian findings. *Journal of Youth and Adolescence,**31*(3), 185–195. 10.1023/A:1015081102189

[CR72] Russel, C., Pang, M., Malta, M., Duarado, L., & Fischer, B. (2023). *A Pre-Release Report Examining Experiences of Federal Offennders on Opioid Agonist Treatment (OAT) During Incarceration in Ontario, Canada: A Pre-Release Report* (No. 449). Correctional Serivce of Canada. https://publications.gc.ca/collections/collection_2023/scc-csc/PS83-3-449-eng.pdf

[CR73] Samuels-Wortley, K. (2022). Black on Blue, Will Not Do: Navigating Canada’s Evidence Based Policing Community as a Black Academic – A Personal Counter-story. In *Diversity in Criminology and Criminal Justice Studies* (world; Vol. 27, pp. 63–80). Emerald Publishing Limited. 10.1108/S1521-613620220000027005

[CR74] Sibblis, C., Delia Deckard, N., & Salawu Anazodo, K. (2022). The colour of system avoidance in Canada: Investigating the importance of immigrant generation among African Canadians. *Canadian Review of Sociology = Revue Canadienne De Sociologie,**59*(4), 470–489. 10.1111/cars.12407 36303418 10.1111/cars.12407

[CR75] Statistics Canada (2015, April 22). *Youth correctional statistics in Canada, 2013/2014*. Government of Canada. https://www150.statcan.gc.ca/n1/pub/85-002-x/2015001/article/14164-eng.htm

[CR76] Syed, I. M., Wilson, C. L., McKie, R., Marcotte, A.-A., & Travers, R. (2018). More threatened than safe: What African, Caribbean, and Black Youth Living In Southern Ontario Say About Their Interactions with Law Enforcement. *Community Psychology in Global Perspective,**4*(2), 101–118. 10.1285/i24212113v4i2p101

[CR77] Tricco, A. C., Lillie, E., Zarin, W., O’Brien, K. K., Colquhoun, H., Levac, D., Moher, D., Peters, M. D. J., Horsley, T., Weeks, L., Hempel, S., Akl, E. A., Chang, C., McGowan, J., Stewart, L., Hartling, L., Aldcroft, A., Wilson, M. G., Garritty, C., & Straus, S. E. (2018). PRISMA extension for scoping reviews (PRISMA-ScR): Checklist and explanation. *Annals of Internal Medicine*, *169*(7), 467–473. 10.7326/M18-0850 30178033 10.7326/M18-0850

[CR78] United Nations General Assembly (2015). *Transforming our world: The 2030 Agenda for Sustainable Development*. https://docs.un.org/en/A/RES/70/1

[CR79] Waldron, I., Senger, B., Cookey, J., Crown, M., & Tibbo, P. (2023). Addressing stigma and promoting help-seeking among African Nova Scotian youth experiencing psychosis and other mental health problems. *Canadian Journal of Psychiatry = Revue Canadienne De Psychiatrie,**68*(3), 200–207. 10.1177/07067437221125305 36113102 10.1177/07067437221125305 PMC9974652

[CR80] Walker, M. (2022). Race and prison. *THE SOCIETY: Sociology and Criminology Undergraduate Review*, *7*(1), Article1. https://jps.library.utoronto.ca/index.php/society/article/view/38494

[CR81] Weldon, I., & Hoffman, S. J. (2021). Harnessing Canada’s Potential for Global Health Leadership: Leveraging Strengths and Confronting Demons. *The Palgrave Handbook of Canada in International Affairs* (pp. 483–510). Palgrave Macmillan. 10.1007/978-3-030-67770-1_22

[CR82] Whitley, R., Wang, J., Fleury, M. J., Liu, A., & Caron, J. (2017). Mental health Status, health care Utilisation, and service satisfaction among immigrants in montreal: An epidemiological comparison. *Canadian Journal of Psychiatry Revue Canadienne De Psychiatrie*, *62*(8), 570–579. 10.1177/0706743716677724 27836931 10.1177/0706743716677724 PMC5546664

[CR83] Williams, K. K. A., Lofters, A., Baidoobonso, S., Leblanc, I., Haggerty, J., & Adams, A. M. (2024). Embracing black heterogeneity: The importance of intersectionality in research on anti-Black racism and health care equity in Canada. *Canadian Medical Association Journal: CMAJ*, *196*(22), E767–E769. 10.1503/cmaj.230350 38857933 10.1503/cmaj.230350 PMC11173650

[CR84] Wortley, S. (2019). *Halifax, Nova Scotia: Street Checks Report* (p. 180). Nova Scotia Human Rights Commission. https://humanrights.novascotia.ca/sites/default/files/editor-uploads/halifax_street_checks_report_march_2019_0.pdf

[CR85] Wortley, S., & Jung, M. (2020). *Racial Disparity in Arrests and Charges* (p. 118). Government of Ontario. https://www.ohrc.on.ca/sites/default/files/Racial%20Disparity%20in%20Arrests%20and%20Charges%20TPS.pdf

[CR86] Wortley, S., & Tanner, J. (2005). Inflammatory rhetoric? Baseless accusations? A response to gabor’s critique of Racial profiling research in Canada. *Canadian Journal of Criminology & Criminal Justice*, *47*(3), 581–609. 10.3138/cjccj.47.3.581

