# Supplementary material for: Insidious systems: a scoping review of Black youth and the criminal justice system in Canada
Source: Health Justice. 2026 Jan 15;14:6. doi: 10.1186/s40352-026-00396-2 (PMC12892758; doi:10.1186/s40352-026-00396-2)
Supplement: Supplementary file 2 — Supplementary Material 2. [file 40352_2026_396_MOESM2_ESM.docx]

**Ovid MEDLINE(R) ALL <1946 to May 20, 2024>**

[**https://ezproxy.lib.ucalgary.ca/login?url=https://ovidsp.ovid.com/ovidweb.cgi?T=JS&NEWS=N&PAGE=main&SHAREDSEARCHID=5aU8Y3ExJQU0iTcX5kupzZg8LHXahdeWGgnElAAeixyS3KXo2zOBXBUlqBUIVQ69A**](https://ezproxy.lib.ucalgary.ca/login?url=https://ovidsp.ovid.com/ovidweb.cgi?T=JS&NEWS=N&PAGE=main&SHAREDSEARCHID=5aU8Y3ExJQU0iTcX5kupzZg8LHXahdeWGgnElAAeixyS3KXo2zOBXBUlqBUIVQ69A)

1 exp Canada/ 186764

2 Canad*.tw,kf. 170576

3 exp British Columbia/ 11730

4 British Columbia.tw,kf. 11514

5 exp Alberta/ 9550

6 Alberta*.tw,kf. 12411

7 exp Saskatchewan/ 2806

8 Saskatchewan.tw,kf. 3574

9 exp Manitoba/ 3628

10 Manitoba*.tw,kf. 4671

11 exp Ontario/ 32650

12 Ontari*.tw,kf. 37348

13 exp New Brunswick/ 797

14 New Brunswick.tw,kf. 1449

15 exp Quebec/ 15356

16 Quebe*.tw,kf. 16063

17 exp Prince Edward Island/ 326

18 Prince Edward Island.tw,kf. 645

19 PEI.tw,kf. 10644

20 exp Nova Scotia/ 2736

21 Nova Scotia*.tw,kf. 3196

22 exp "Newfoundland and Labrador"/ 1508

23 Newfoundland.tw,kf. 2072

24 exp Yukon Territory/ 253

25 Yukon.tw,kf. 787

26 exp Northwest Territories/ 443

27 Northwest Territories.tw,kf. 665

28 NWT.tw,kf. 294

29 exp Nunavut/ 384

30 Nunavut.tw,kf. 669

31 1 or 2 or 3 or 4 or 5 or 6 or 7 or 8 or 9 or 10 or 11 or 12 or 13 or 14 or 15 or 16 or 17 or 18 or 19 or 20 or 21 or 22 or 23 or 24 or 25 or 26 or 27 or 28 or 29 or 30 293894

32 exp Algeria/ 3642

33 Algeri*.tw,kf. 6013

34 exp Angola/ 1179

35 Angol*.tw,kf. 2354

36 exp Benin/ 2003

37 Benin.tw,kf. 4353

38 exp Botswana/ 2255

39 Botswana.tw,kf. 3174

40 exp Burkina Faso/ 4136

41 Burkina Faso.tw,kf. 5431

42 exp Burundi/ 732

43 Burundi.tw,kf. 1098

44 exp Cabo Verde/ 266

45 Cabo Verde.tw,kf. 190

46 exp Cameroon/ 6772

47 Cameroon.tw,kf. 9091

48 exp Central African Republic/ 858

49 Central African Republic.tw,kf. 1226

50 exp Chad/ 846

51 Chad.tw,kf. 1602

52 exp Comoros/ 389

53 Comoros.tw,kf. 467

54 exp "Democratic Republic of the Congo"/ 5227

55 Democratic Republic of the Congo.tw,kf. 4533

56 Congo*.tw,kf. 20113

57 exp Djibouti/ 256

58 Djibouti.tw,kf. 478

59 exp Egypt/ 18093

60 Egypt.tw,kf. 19704

61 exp Equatorial Guinea/ 326

62 Equatorial Guinea.tw,kf. 531

63 exp Eritrea/ 433

64 Eritrea.tw,kf. 754

65 exp Eswatini/ 796

66 Eswatini.tw,kf. 420

67 Swaziland.tw,kf. 896

68 exp Ethiopia/ 20856

69 Ethiopia.tw,kf. 30649

70 exp Gabon/ 1665

71 Gabon.tw,kf. 2126

72 exp Gambia/ 2766

73 Gambia*.tw,kf. 9448

74 exp Ghana/ 11893

75 Ghan*.tw,kf. 17855

76 exp Guinea/ 1364

77 exp Guinea-Bissau/ 1049

78 Guinea*.tw,kf. 114236

79 exp Cote d'Ivoire/ 3648

80 Cote d'Ivoire.tw,kf. 2957

81 Ivory Coast.tw,kf. 1974

82 exp Kenya/ 20233

83 Kenya*.tw,kf. 26795

84 exp Lesotho/ 566

85 Lesotho.tw,kf. 1033

86 exp Liberia/ 1441

87 Liberia*.tw,kf. 2228

88 exp Libya/ 1249

89 Libya*.tw,kf. 2160

90 exp Madagascar/ 4010

91 Madagascar.tw,kf. 6027

92 exp Malawi/ 7000

93 Malawi.tw,kf. 9521

94 exp Mali/ 2782

95 Mali.tw,kf. 4689

96 exp Mauritania/ 514

97 Mauritania.tw,kf. 809

98 exp Mauritius/ 634

99 Mauritius.tw,kf. 1230

100 exp Morocco/ 6759

101 Morroc*.tw,kf. 59

102 exp Mozambique/ 3166

103 Mozambique.tw,kf. 4850

104 exp Namibia/ 1355

105 Namibia.tw,kf. 2195

106 exp Niger/ 1481

107 Niger.tw,kf. 15287

108 exp Nigeria/ 35298

109 Nigeria*.tw,kf. 45598

110 exp Rwanda/ 3304

111 Rwanda*.tw,kf. 4688

112 Sao Tome.tw,kf. 343

113 Principe.tw,kf. 540

114 exp Senegal/ 6337

115 Senegal*.tw,kf. 9220

116 exp Seychelles/ 462

117 Seychelles.tw,kf. 928

118 exp Sierra Leone/ 2021

119 Sierra Leone.tw,kf. 3076

120 exp Somalia/ 1966

121 exp South Africa/ 50844

122 South Africa*.tw,kf. 58134

123 exp South Sudan/ 295

124 exp Sudan/ 5270

125 Sudan*.tw,kf. 12413

126 exp Tanzania/ 14446

127 Tanzania.tw,kf. 17477

128 exp Togo/ 1319

129 Togo.tw,kf. 1925

130 exp Tunisia/ 9413

131 Tunisia*.tw,kf. 12787

132 exp Uganda/ 16140

133 Uganda*.tw,kf. 21920

134 exp Zambia/ 5726

135 Zambia*.tw,kf. 7773

136 exp Zimbabwe/ 6725

137 Zimbabwe.tw,kf. 7184

138 exp Bahamas/ 516

139 Baham*.tw,kf. 1113

140 exp Barbados/ 676

141 Barbados.tw,kf. 1138

142 exp Cuba/ 5399

143 Cuba*.tw,kf. 9239

144 exp Dominica/ 116

145 Dominica*.tw,kf. 3856

146 exp Grenada/ 173

147 Grenada.tw,kf. 394

148 exp Haiti/ 3658

149 Haiti*.tw,kf. 4777

150 exp Jamaica/ 3648

151 Jamaica*.tw,kf. 4826

152 Saint Kitts.tw,kf. 67

153 Nevis.tw,kf. 155

154 exp Saint Lucia/ 76

155 Saint Lucia*.tw,kf. 110

156 Saint Vincent.tw,kf. 226

157 exp "Saint Vincent and the Grenadines"/ 59

158 Grenadines.tw,kf. 108

159 exp "Trinidad and Tobago"/ 1937

160 Trinidad.tw,kf. 2719

161 Tobago.tw,kf. 1268

162 exp Anguilla/ 1975

163 Anguilla*.tw,kf. 4450

164 exp "Antigua and Barbuda"/ 116

165 Antigua.tw,kf. 289

166 Barbuda.tw,kf. 94

167 exp Barbados/ 676

168 Barbados.tw,kf. 1138

169 exp Aruba/ 7

170 Aruba.tw,kf. 268

171 Bonaire.tw,kf. 91

172 exp British Virgin Islands/ 6

173 British Virgin Islands.tw,kf. 66

174 exp West Indies/ 29165

175 west indies.tw,kf. 2999

176 Cayman Islands.tw,kf. 114

177 Curacao.tw,kf. 531

178 exp Curacao/ 56

179 exp Guadeloupe/ 566

180 Guadeloupe.tw,kf. 1123

181 exp Martinique/ 633

182 Martinique.tw,kf. 961

183 Montserrat.tw,kf. 147

184 exp Puerto Rico/ 7213

185 Afro-Puerto Rican*.tw,kf. 0

186 Saba.tw,kf. 1037

187 Saint Barth*.tw,kf. 36

188 St Barth*.tw,kf. 540

189 exp Sint Maarten/ 5

190 Eustatius.tw,kf. 40

191 St Martin.tw,kf. 86

192 St Maarten.tw,kf. 21

193 Caicos.tw,kf. 51

194 exp United States Virgin Islands/ 334

195 Virgin Islands.tw,kf. 694

196 exp "Black or African American"/ 63491

197 Black*.tw,kf. 207990

198 Afro-.tw,kf. 3235

199 exp African People/ 246

200 Africa*.tw,kf. 310780

201 exp Caribbean People/ 39

202 Caribbean.tw,kf. 19248

203 exp Black People/ 99058

204 ((person* or people*) adj2 colo?r).tw,kf. 2166

205 BIPOC.tw,kf. 391

206 32 or 33 or 34 or 35 or 36 or 37 or 38 or 39 or 40 or 41 or 42 or 43 or 44 or 45 or 46 or 47 or 48 or 49 or 50 or 51 or 52 or 53 or 54 or 55 or 56 or 57 or 58 or 59 or 60 or 61 or 62 or 63 or 64 or 65 or 66 or 67 or 68 or 69 or 70 or 71 or 72 or 73 or 74 or 75 or 76 or 77 or 78 or 79 or 80 or 81 or 82 or 83 or 84 or 85 or 86 or 87 or 88 or 89 or 90 or 91 or 92 or 93 or 94 or 95 or 96 or 97 or 98 or 99 or 100 or 101 or 102 or 103 or 104 or 105 or 106 or 107 or 108 or 109 or 110 or 111 or 112 or 113 or 114 or 115 or 116 or 117 or 118 or 119 or 120 or 121 or 122 or 123 or 124 or 125 or 126 or 127 or 128 or 129 or 130 or 131 or 132 or 133 or 134 or 135 or 136 or 137 or 138 or 139 or 140 or 141 or 142 or 143 or 144 or 145 or 146 or 147 or 148 or 149 or 150 or 151 or 152 or 153 or 154 or 155 or 156 or 157 or 158 or 159 or 160 or 161 or 162 or 163 or 164 or 165 or 166 or 167 or 168 or 169 or 170 or 171 or 172 or 173 or 174 or 175 or 176 or 177 or 178 or 179 or 180 or 181 or 182 or 183 or 184 or 185 or 186 or 187 or 188 or 189 or 190 or 191 or 192 or 193 or 194 or 195 or 196 or 197 or 198 or 199 or 200 or 201 or 202 or 203 or 204 or 205 977264

207 ((young* or youth* or teen* or adolescen* or child* or p?ediatric*) adj2 (man or men or male* or woman or women or female* or person* or people* or Black)).tw,kf. 264745

208 exp Adolescent/ 2247979

209 exp Pediatrics/ 63515

210 exp Child/ 2205579

211 exp Infant/ 1275314

212 exp Infant, Newborn/ 687501

213 adolescen*.tw,kf. 393397

214 p?ediatric*.tw,kf. 499842

215 child*.tw,kf. 1746841

216 infant*.tw,kf. 520879

217 newborn*.tw,kf. 204023

218 teen*.tw,kf. 37206

219 youth*.tw,kf. 111692

220 207 or 208 or 209 or 210 or 211 or 212 or 213 or 214 or 215 or 216 or 217 or 218 or 219 4859152

221 31 and 206 and 220 1682

**Embase <1974 to 2024 May 20>**

[**https://ezproxy.lib.ucalgary.ca/login?url=https://ovidsp.ovid.com/ovidweb.cgi?T=JS&NEWS=N&PAGE=main&SHAREDSEARCHID=2aLCntAMQb4YbjGfTteNroNm0KMp1kEKEAknvFppBrbr5i4NC8bmK3f6xKeQ5EWFf**](https://ezproxy.lib.ucalgary.ca/login?url=https://ovidsp.ovid.com/ovidweb.cgi?T=JS&NEWS=N&PAGE=main&SHAREDSEARCHID=2aLCntAMQb4YbjGfTteNroNm0KMp1kEKEAknvFppBrbr5i4NC8bmK3f6xKeQ5EWFf)

1 exp Canada/ 223278

2 Canad*.tw,kf. 237596

3 exp British Columbia/ 5434

4 British Columbia.tw,kf. 15028

5 exp Alberta/ 3525

6 Alberta*.tw,kf. 17075

7 exp Saskatchewan/ 1244

8 Saskatchewan.tw,kf. 4139

9 exp Manitoba/ 1776

10 Manitoba*.tw,kf. 5863

11 exp Ontario/ 14486

12 Ontari*.tw,kf. 48870

13 exp New Brunswick/ 510

14 New Brunswick.tw,kf. 1717

15 exp Quebec/ 5633

16 Quebe*.tw,kf. 20651

17 exp Prince Edward Island/ 232

18 Prince Edward Island.tw,kf. 683

19 PEI.tw,kf. 13199

20 exp Nova Scotia/ 1337

21 Nova Scotia*.tw,kf. 4156

22 exp "Newfoundland and Labrador"/ 747

23 Newfoundland.tw,kf. 2451

24 exp Yukon Territory/ 202

25 Yukon.tw,kf. 826

26 exp Northwest Territories/ 184

27 Northwest Territories.tw,kf. 728

28 NWT.tw,kf. 387

29 exp Nunavut/ 242

30 Nunavut.tw,kf. 727

31 1 or 2 or 3 or 4 or 5 or 6 or 7 or 8 or 9 or 10 or 11 or 12 or 13 or 14 or 15 or 16 or 17 or 18 or 19 or 20 or 21 or 22 or 23 or 24 or 25 or 26 or 27 or 28 or 29 or 30 364233

32 exp Algeria/ 5981

33 Algeri*.tw,kf. 8779

34 exp Angola/ 1840

35 Angol*.tw,kf. 2573

36 exp Benin/ 3338

37 Benin.tw,kf. 5822

38 exp Botswana/ 3486

39 Botswana.tw,kf. 3723

40 exp Burkina Faso/ 5680

41 Burkina Faso.tw,kf. 6461

42 exp Burundi/ 1124

43 Burundi.tw,kf. 1188

44 exp Cabo Verde/ 569

45 Cabo Verde.tw,kf. 185

46 exp Cameroon/ 9223

47 Cameroon.tw,kf. 10508

48 exp Central African Republic/ 1051

49 Central African Republic.tw,kf. 1301

50 exp Chad/ 996

51 Chad.tw,kf. 1919

52 exp Comoros/ 452

53 Comoros.tw,kf. 472

54 exp "Democratic Republic of the Congo"/ 5131

55 Democratic Republic of the Congo.tw,kf. 5358

56 Congo*.tw,kf. 24029

57 exp Djibouti/ 439

58 Djibouti.tw,kf. 555

59 exp Egypt/ 25299

60 Egypt.tw,kf. 27478

61 exp Equatorial Guinea/ 612

62 Equatorial Guinea.tw,kf. 706

63 exp Eritrea/ 716

64 Eritrea.tw,kf. 858

65 exp Eswatini/ 385

66 Eswatini.tw,kf. 487

67 Swaziland.tw,kf. 1125

68 exp Ethiopia/ 29455

69 Ethiopia.tw,kf. 30799

70 exp Gabon/ 2049

71 Gabon.tw,kf. 2435

72 exp Gambia/ 3344

73 Gambia*.tw,kf. 10723

74 exp Ghana/ 16933

75 Ghan*.tw,kf. 20393

76 exp Guinea/ 2536

77 exp Guinea-Bissau/ 1217

78 Guinea*.tw,kf. 108300

79 exp Cote d'Ivoire/ 3848

80 Cote d'Ivoire.tw,kf. 3484

81 Ivory Coast.tw,kf. 2165

82 exp Kenya/ 26712

83 Kenya*.tw,kf. 30806

84 exp Lesotho/ 1051

85 Lesotho.tw,kf. 1160

86 exp Liberia/ 2079

87 Liberia*.tw,kf. 2405

88 exp Libya/ 2056

89 Libya*.tw,kf. 2660

90 exp Madagascar/ 5289

91 Madagascar.tw,kf. 6409

92 exp Malawi/ 10229

93 Malawi.tw,kf. 11075

94 exp Mali/ 4352

95 Mali.tw,kf. 5614

96 exp Mauritania/ 821

97 Mauritania.tw,kf. 879

98 exp Mauritius/ 1096

99 Mauritius.tw,kf. 1285

100 exp Morocco/ 9837

101 Morroc*.tw,kf. 161

102 exp Mozambique/ 5064

103 Mozambique.tw,kf. 5587

104 exp Namibia/ 2179

105 Namibia.tw,kf. 2386

106 exp Niger/ 2770

107 Niger.tw,kf. 19491

108 exp Nigeria/ 47604

109 Nigeria*.tw,kf. 55494

110 exp Rwanda/ 5182

111 Rwanda*.tw,kf. 5549

112 Sao Tome.tw,kf. 361

113 Principe.tw,kf. 586

114 exp Senegal/ 7460

115 Senegal*.tw,kf. 10767

116 exp Seychelles/ 598

117 Seychelles.tw,kf. 955

118 exp Sierra Leone/ 3158

119 Sierra Leone.tw,kf. 3434

120 exp Somalia/ 2639

121 exp South Africa/ 62118

122 South Africa*.tw,kf. 67865

123 exp South Sudan/ 583

124 exp Sudan/ 7551

125 Sudan*.tw,kf. 14148

126 exp Tanzania/ 19925

127 Tanzania.tw,kf. 20269

128 exp Togo/ 1825

129 Togo.tw,kf. 2194

130 exp Tunisia/ 12829

131 Tunisia*.tw,kf. 18931

132 exp Uganda/ 22553

133 Uganda*.tw,kf. 25294

134 exp Zambia/ 8257

135 Zambia*.tw,kf. 9067

136 exp Zimbabwe/ 7971

137 Zimbabwe.tw,kf. 7747

138 exp Bahamas/ 849

139 Baham*.tw,kf. 1352

140 exp Barbados/ 1000

141 Barbados.tw,kf. 1358

142 exp Cuba/ 7642

143 Cuba*.tw,kf. 11869

144 exp Dominica/ 230

145 Dominica*.tw,kf. 4815

146 exp Grenada/ 362

147 Grenada.tw,kf. 471

148 exp Haiti/ 4884

149 Haiti*.tw,kf. 6001

150 exp Jamaica/ 4034

151 Jamaica*.tw,kf. 5367

152 Saint Kitts.tw,kf. 65

153 Nevis.tw,kf. 189

154 exp Saint Lucia/ 137

155 Saint Lucia*.tw,kf. 96

156 Saint Vincent.tw,kf. 258

157 exp "Saint Vincent and the Grenadines"/ 101

158 Grenadines.tw,kf. 132

159 exp "Trinidad and Tobago"/ 1896

160 Trinidad.tw,kf. 3014

161 Tobago.tw,kf. 1551

162 exp Anguilla/ 0

163 Anguilla*.tw,kf. 4559

164 exp "Antigua and Barbuda"/ 186

165 Antigua.tw,kf. 262

166 Barbuda.tw,kf. 100

167 exp Barbados/ 1000

168 Barbados.tw,kf. 1358

169 exp Aruba/ 124

170 Aruba.tw,kf. 348

171 Bonaire.tw,kf. 98

172 exp British Virgin Islands/ 53

173 British Virgin Islands.tw,kf. 61

174 exp West Indies/ 36203

175 west indies.tw,kf. 3209

176 Cayman Islands.tw,kf. 124

177 Curacao.tw,kf. 739

178 exp Curacao/ 212

179 exp Guadeloupe/ 627

180 Guadeloupe.tw,kf. 1211

181 exp Martinique/ 742

182 Martinique.tw,kf. 1120

183 Montserrat.tw,kf. 183

184 exp Puerto Rico/ 8631

185 Afro-Puerto Rican*.tw,kf. 2

186 Saba.tw,kf. 2148

187 Saint Barth*.tw,kf. 36

188 St Barth*.tw,kf. 464

189 exp Sint Maarten/ 78

190 Eustatius.tw,kf. 33

191 St Martin.tw,kf. 95

192 St Maarten.tw,kf. 27

193 Caicos.tw,kf. 59

194 exp United States Virgin Islands/ 339

195 Virgin Islands.tw,kf. 733

196 exp "Black or African American"/ 116028

197 Black*.tw,kf. 364651

198 Afro-.tw,kf. 4960

199 exp African People/ 42687

200 Africa*.tw,kf. 386250

201 exp Caribbean People/ 3462

202 Caribbean.tw,kf. 22449

203 exp Black People/ 152930

204 ((person* or people*) adj2 colo?r).tw,kf. 2545

205 BIPOC.tw,kf. 512

206 32 or 33 or 34 or 35 or 36 or 37 or 38 or 39 or 40 or 41 or 42 or 43 or 44 or 45 or 46 or 47 or 48 or 49 or 50 or 51 or 52 or 53 or 54 or 55 or 56 or 57 or 58 or 59 or 60 or 61 or 62 or 63 or 64 or 65 or 66 or 67 or 68 or 69 or 70 or 71 or 72 or 73 or 74 or 75 or 76 or 77 or 78 or 79 or 80 or 81 or 82 or 83 or 84 or 85 or 86 or 87 or 88 or 89 or 90 or 91 or 92 or 93 or 94 or 95 or 96 or 97 or 98 or 99 or 100 or 101 or 102 or 103 or 104 or 105 or 106 or 107 or 108 or 109 or 110 or 111 or 112 or 113 or 114 or 115 or 116 or 117 or 118 or 119 or 120 or 121 or 122 or 123 or 124 or 125 or 126 or 127 or 128 or 129 or 130 or 131 or 132 or 133 or 134 or 135 or 136 or 137 or 138 or 139 or 140 or 141 or 142 or 143 or 144 or 145 or 146 or 147 or 148 or 149 or 150 or 151 or 152 or 153 or 154 or 155 or 156 or 157 or 158 or 159 or 160 or 161 or 162 or 163 or 164 or 165 or 166 or 167 or 168 or 169 or 170 or 171 or 172 or 173 or 174 or 175 or 176 or 177 or 178 or 179 or 180 or 181 or 182 or 183 or 184 or 185 or 186 or 187 or 188 or 189 or 190 or 191 or 192 or 193 or 194 or 195 or 196 or 197 or 198 or 199 or 200 or 201 or 202 or 203 or 204 or 205 1266586

207 ((young* or youth* or teen* or adolescen* or child* or p?ediatric*) adj2 (man or men or male* or woman or women or female* or person* or people* or Black)).tw,kf. 351183

208 exp Adolescent/ 1841250

209 exp Pediatrics/ 130487

210 exp Child/ 3201404

211 exp Infant/ 1171397

212 exp Infant, Newborn/ 620689

213 adolescen*.tw,kf. 498807

214 p?ediatric*.tw,kf. 784876

215 child*.tw,kf. 2196992

216 infant*.tw,kf. 566533

217 newborn*.tw,kf. 233909

218 teen*.tw,kf. 51877

219 youth*.tw,kf. 131790

220 207 or 208 or 209 or 210 or 211 or 212 or 213 or 214 or 215 or 216 or 217 or 218 or 219 5127323

221 31 and 206 and 220 2583

**APA PsycInfo <1806 to May Week 3 2024>**

<https://ezproxy.lib.ucalgary.ca/login?url=https://ovidsp.ovid.com/ovidweb.cgi?T=JS&NEWS=N&PAGE=main&SHAREDSEARCHID=1CFH7OR0nAppj7IHdTWjbedaF15LzBrAnl4PUDB4fx9b4JDgV36qu81v5R8jrz7CS>

1 Canad*.tw,id. 59455

2 British Columbia.tw,id. 2692

3 Alberta*.tw,id. 2356

4 Saskatchewan.tw,id. 581

5 Manitoba*.tw,id. 904

6 Ontario*.tw,id. 8510

7 New Brunswick.tw,id. 296

8 Quebe*.tw,id. 4581

9 Prince Edward Island.tw,id. 84

10 PEI.tw,id. 452

11 Nova Scotia*.tw,id. 657

12 Newfoundland.tw,id. 411

13 Yukon.tw,id. 87

14 Northwest Territor*.tw,id. 103

15 NWT.tw,id. 36

16 Nunavut.tw,id. 121

17 1 or 2 or 3 or 4 or 5 or 6 or 7 or 8 or 9 or 10 or 11 or 12 or 13 or 14 or 15 or 16 67853

18 Algeria*.tw,id. 634

19 Angol*.tw,id. 447

20 Benin.tw,id. 349

21 Botswana.tw,id. 994

22 Burkina Faso.tw,id. 414

23 Burundi.tw,id. 179

24 Cabo Verde.tw,id. 13

25 Cameroon.tw,id. 795

26 Central African Republic.tw,id. 93

27 Chad.tw,id. 377

28 Comoros.tw,id. 12

29 Congo*.tw,id. 1319

30 Djibouti.tw,id. 31

31 Egypt.tw,id. 2680

32 Guinea.tw,id. 4615

33 Eritrea.tw,id. 154

34 Eswatini.tw,id. 75

35 Swaziland.tw,id. 269

36 Ethiopia*.tw,id. 3158

37 Gabon.tw,id. 131

38 Gambia*.tw,id. 288

39 Ghana.tw,id. 3786

40 Cote d'Ivoire.tw,id. 287

41 Ivory Coast.tw,id. 227

42 Kenya*.tw,id. 4819

43 Lesotho.tw,id. 283

44 Liberia*.tw,id. 545

45 Libya*.tw,id. 318

46 Madagascar.tw,id. 615

47 Malawi.tw,id. 1389

48 Mali.tw,id. 382

49 Mauritania.tw,id. 64

50 Mauritius.tw,id. 254

51 Morroc*.tw,id. 15

52 Mozambique.tw,id. 645

53 Namibia.tw,id. 484

54 Niger.tw,id. 478

55 Nigeria*.tw,id. 7904

56 Rwanda*.tw,id. 1330

57 Sao Tome.tw,id. 19

58 Principe.tw,id. 253

59 Senegal*.tw,id. 894

60 Seychelles.tw,id. 81

61 Sierra Leone.tw,id. 600

62 Somalia*.tw,id. 481

63 South Africa*.tw,id. 17546

64 Sudan*.tw,id. 1002

65 Tanzania.tw,id. 2608

66 Togo.tw,id. 191

67 Tunisia.tw,id. 703

68 Uganda*.tw,id. 4074

69 Zambia.tw,id. 1191

70 Zimbabwe.tw,id. 1539

71 Baham*.tw,id. 285

72 Barbados.tw,id. 344

73 Cuba*.tw,id. 2438

74 Dominica*.tw,id. 1386

75 Grenada*.tw,id. 117

76 Haiti*.tw,id. 1636

77 Jamaica*.tw,id. 1640

78 Saint Kitts.tw,id. 5

79 Nevis.tw,id. 76

80 Saint Lucia.tw,id. 23

81 Saint Vincent.tw,id. 28

82 Grenadines.tw,id. 37

83 Trinidad.tw,id. 816

84 Tobago.tw,id. 471

85 Anguilla.tw,id. 39

86 Antigua.tw,id. 84

87 Barbuda.tw,id. 19

88 Barbados.tw,id. 344

89 Aruba.tw,id. 31

90 Bonaire.tw,id. 16

91 British Virgin Islands.tw,id. 16

92 West Indies.tw,id. 366

93 Caymen.tw,id. 0

94 Curacao.tw,id. 80

95 Guadeloupe.tw,id. 54

96 Martinique.tw,id. 85

97 Puerto-Ric*.tw,id. 5282

98 Saba.tw,id. 155

99 Saint Barth*.tw,id. 4

100 St Barth*.tw,id. 26

101 Sint Maarten.tw,id. 5

102 Eustatius.tw,id. 6

103 St Martin.tw,id. 10

104 St Maarten.tw,id. 7

105 Caicos.tw,id. 4

106 Virgin Islands.tw,id. 226

107 exp Blacks/ 62629

108 Black*.tw,id. 87911

109 Afro-.tw,id. 1659

110 exp African Cultural Groups/ 4293

111 exp "Racial and Ethnic Groups"/ 162101

112 Africa*.tw,id. 98071

113 exp Caribbean Cultural Groups/ 642

114 ((person* or people*) adj2 colo?r).tw,id. 3705

115 exp "People of Color"/ 1242

116 BIPOC.tw,id. 470

117 18 or 19 or 20 or 21 or 22 or 23 or 24 or 25 or 26 or 27 or 28 or 29 or 30 or 31 or 32 or 33 or 34 or 35 or 36 or 37 or 38 or 39 or 40 or 41 or 42 or 43 or 44 or 45 or 46 or 47 or 48 or 49 or 50 or 51 or 52 or 53 or 54 or 55 or 56 or 57 or 58 or 59 or 60 or 61 or 62 or 63 or 64 or 65 or 66 or 67 or 68 or 69 or 70 or 71 or 72 or 73 or 74 or 75 or 76 or 77 or 78 or 79 or 80 or 81 or 82 or 83 or 84 or 85 or 86 or 87 or 88 or 89 or 90 or 91 or 92 or 93 or 94 or 95 or 96 or 97 or 98 or 99 or 100 or 101 or 102 or 103 or 104 or 105 or 106 or 107 or 108 or 109 or 110 or 111 or 112 or 113 or 114 or 115 or 116 307839

118 exp Adolescent Behavior/ or exp Adolescent Health/ 9194

119 ((young* or youth* or teen* or adolescen* or child* or p?ediatric*) adj2 (man or men or male* or woman or women or female* or person* or people* or Black)).tw,id. 137927

120 exp Pediatrics/ 38636

121 p?ediatric*.tw,id. 50321

122 exp Child Behavior/ or exp Child Health/ 2881

123 child*.tw,id. 824856

124 infant*.tw,id. 94726

125 newborn*.tw,id. 12993

126 teen*.tw,id. 26683

127 youth.tw,id. 118919

128 118 or 119 or 120 or 121 or 122 or 123 or 124 or 125 or 126 or 127 1053026

129 17 and 117 and 128 1700

**Academic Search Complete** 513

black or African or Caribbean or Trinidad or Jamaican or afro- or color or colour or bipoc [abstract]

AND

child* OR pediatric* Or paediatric* OR youth or adolescents or young people or teen or young adults [abstract]

AND

canad* or British Columbia or alberta or Saskatchewan or Manitoba or Ontario or Quebec or new Brunswick or prince Edward island or nova scotia or newfoundland or Yukon or northwest territories or Nunavut [abstract]

Limit to academic journals

**SocINDEX with Full Text** 248

black or African or Caribbean or Trinidad or Jamaican or afro- or color or colour or bipoc [abstract]

AND

child* OR pediatric* Or paediatric* OR youth or adolescents or young people or teen or young adults [abstract]

AND

canad* or British Columbia or alberta or Saskatchewan or Manitoba or Ontario or Quebec or new Brunswick or prince Edward island or nova scotia or newfoundland or Yukon or northwest territories or Nunavut [abstract]

Limit to academic journals

**Web of Science** 736

https://www.webofscience.com/wos/woscc/summary/898c0c83-3cc3-4ff3-883a-d8d8335e0a11-ea6ca25d/relevance/1

black or African or Caribbean or Trinidad or Jamaican or afro- or color or colour or bipoc [abstract]

AND

child* OR pediatric* Or paediatric* OR youth or adolescents or young people or teen or young adults [abstract]

AND

canad* or British Columbia or alberta or Saskatchewan or Manitoba or Ontario or Quebec or new Brunswick or prince Edward island or nova scotia or newfoundland or Yukon or northwest territories or Nunavut [abstract]

**Scopus** 616

black or African or Caribbean or Trinidad or Jamaican or afro- or color or colour or bipoc [article title,abstract, keywords]

AND

child* OR pediatric* Or paediatric* OR youth or adolescents or young people or teen or young adults [article title, abstract, keywords]

AND

canad* or British Columbia or alberta or Saskatchewan or Manitoba or Ontario or Quebec or new Brunswick or prince Edward island or nova scotia or newfoundland or Yukon or northwest territories or Nunavut [article title, abstract, keywords]
